# The Cdk8/19-cyclin C transcription regulator functions in genome replication through metazoan Sld7

**DOI:** 10.1371/journal.pbio.2006767

**Published:** 2019-01-29

**Authors:** Kerstin Köhler, Luis Sanchez-Pulido, Verena Höfer, Anika Marko, Chris P. Ponting, Ambrosius P. Snijders, Regina Feederle, Aloys Schepers, Dominik Boos

**Affiliations:** 1 Vertebrate DNA Replication Lab, Centre for Medical Biotechnology, University of Duisburg-Essen, Essen, Germany; 2 Medical Research Council Human Genetics Unit, IGMM, University of Edinburgh, Edinburgh, Scotland, United Kingdom; 3 Mass Spectrometry Proteomics Science Technology Platform, The Francis Crick Institute, London, United Kingdom; 4 Monoclonal Antibody Core Facility and Research Group, Helmholtz Zentrum, Munich GmbH; Institute for Diabetes and Obesity, Neuherberg, Germany; 5 Department of Gene Vectors, Helmholtz Zentrum München GmbH, Munich, Germany; Memorial Sloan Kettering Cancer Center, United States of America

## Abstract

Accurate genome duplication underlies genetic homeostasis. Metazoan Mdm2 binding protein (MTBP) forms a main regulatory platform for origin firing together with Treslin/TICRR and TopBP1 (Topoisomerase II binding protein 1 (TopBP1)–interacting replication stimulating protein/TopBP1-interacting checkpoint and replication regulator). We report the first comprehensive analysis of MTBP and reveal conserved and metazoa-specific MTBP functions in replication. This suggests that metazoa have evolved specific molecular mechanisms to adapt replication principles conserved with yeast to the specific requirements of the more complex metazoan cells. We uncover one such metazoa-specific process: a new replication factor, cyclin-dependent kinase 8/19–cyclinC (Cdk8/19-cyclin C), binds to a central domain of MTBP. This interaction is required for complete genome duplication in human cells. In the absence of MTBP binding to Cdk8/19-cyclin C, cells enter mitosis with incompletely duplicated chromosomes, and subsequent chromosome segregation occurs inaccurately. Using remote homology searches, we identified MTBP as the metazoan orthologue of yeast synthetic lethal with Dpb11 7 (Sld7). This homology finally demonstrates that the set of yeast core factors sufficient for replication initiation in vitro is conserved in metazoa. MTBP and Sld7 contain two homologous domains that are present in no other protein, one each in the N and C termini. In MTBP the conserved termini flank the metazoa-specific Cdk8/19-cyclin C binding region and are required for normal origin firing in human cells. The N termini of MTBP and Sld7 share an essential origin firing function, the interaction with Treslin/TICRR or its yeast orthologue Sld3, respectively. The C termini may function as homodimerisation domains. Our characterisation of broadly conserved and metazoa-specific initiation processes sets the basis for further mechanistic dissection of replication initiation in vertebrates. It is a first step in understanding the distinctions of origin firing in higher eukaryotes.

## Author summary

Efficient and well-regulated DNA replication origin firing is central to ensure complete and accurate genome duplication before cell division. We here use bioinformatics and cultured human cells to understand the role of the essential origin firing factor Mdm2 binding protein (MTBP). We prove that MTBP is orthologous to yeast Sld7. Their homologous N-terminal domains bind the orthologous firing factors Treslin/TICRR and Sld3, respectively. The homologous C termini may constitute MTBP/Sld7 homodimerisation domains. Because Sld7 was the only yeast core origin firing factor for which no metazoan orthologue had been found, our work proves that the complement of core firing factors is conserved from yeast to man. We also find that MTBP contains a central region that is not found in yeast, and that is important for replication in human cells, showing that—despite fundamental conservation—higher eukaryotes have also evolved specific origin firing processes. The Cdk8/19-cyclin C transcription kinase that has not previously been implicated in DNA replication binds the central MTBP region, and this is required for complete DNA replication and proper chromosome segregation in human cells. Our work suggests that MTBP helps integrate universal conserved eukaryotic origin firing processes into the complex metazoan cells.

## Introduction

Eukaryotic cells must faithfully replicate their genomic DNA exactly once before each cell division in order to ensure genetic homeostasis through successive cell generations. Initiation of DNA replication is a major step of replication control by the cell cycle and the DNA damage checkpoint, ensuring faithful genome duplication in normal and adverse conditions [1, 2]. In higher eukaryotic cells, we know little about the molecular mechanisms and regulation of replication initiation. Yeast cells have served as a model for understanding initiation in eukaryotes. In the first initiation step, origin licensing, that occurs in the G1 cell cycle phase, prereplicative complexes (pre-RCs) form dependently on the origin recognition complex (ORC), cell division cycle 6 (Cdc6), and Cdc10-dependent transcript (Cdt1). Pre-RCs are inactive double hexamers of the Mcm2-7 helicase, comprising the six minichromosome maintenance (Mcm) proteins 2–7, loaded on origin DNA. In S phase, high activity of S phase cyclin-dependent kinase (S-CDK) and dumbbell former 4 (Dbf4)–dependent kinase (DDK) induces origin firing, the conversion of pre-RCs into two bidirectional replisomes, each containing a Mcm2-7 hexamer, Cdc45, and the go-ichi-ni-san (GINS) complex. This Cdc45-Mcm2-7-GINS (CMG) complex is the active replicative helicase [3]. DDK phosphorylates pre-RCs, which recruits the synthetic lethal with Dpb11 3/7 (Sld3-Sld7) complex to pre-RCs, which in turn interacts with and recruits Cdc45 [4–6]. S-CDK facilitates origin firing by phosphorylating Sld3 and Sld2 [7–9]. Phospho-Sld3 interacts with DNA polymerase binding 11 (Dpb11), which is part of the preloading complex (pre-LC) [10]. This interaction is thought to locate pre-LCs at origins. Pre-LCs also contain Sld2, polymerase epsilon, and the GINS tetramer. Pre-LC formation depends on S-CDK that phosphorylates Sld2 to induce the interaction of Sld2 with Dpb11 [11]. Formation of active replisomes further requires Mcm10, whose function is less clear [12, 13].

Biochemical reconstitution demonstrated that these yeast core initiation factors are sufficient to initiate bidirectional DNA replication [14–16]. Metazoan orthologues for each of the yeast core factors have previously been identified, except for Sld7. *Sld7*-deleted cells are viable but have severe replication defects [17]. In vitro replication initiates in the absence of Sld7 [6]. Whether the dispensability of Sld7 in vitro reflects an indirect role of Sld7 in origin firing in vivo or whether it is a consequence of the special biochemical in vitro conditions is unclear. Sld7 forms a stable complex with Sld3 in vivo and in vitro [17, 18]. Yeast cells require the interaction of Sld7 with Sld3 to replicate efficiently, because Sld7 binding mutants of Sld3 are sensitive to hydroxyurea, just like *sld7Δ* cells [17].

Conservation of most, but potentially not all, origin firing factors between yeast and metazoa suggested that although many fundamental initiation processes are conserved, processes that are specific for higher eukaryotes might have evolved. All metazoan orthologues of core yeast firing factors are essential for replication, as studies in *Xenopus* egg extracts and human cells have shown, but their molecular functions are largely unexplored [19–24]. The Sld3 orthologue, Treslin/TICRR (Topoisomerase II binding protein 1 (TopBP1)–interacting replication stimulating protein/TopBP1-interacting checkpoint and replication regulator) [25], utilises conserved domains for S-CDK–dependent interaction with the Dpb11 orthologue TopBP1 [24, 26, 27].

The Mdm2 binding protein (MTBP) protein was the last metazoan firing factor identified and described to be required for firing in human cells [28]. It did not fit a universal model of eukaryotic replication because, despite our extensive efforts, no homology with yeast initiation proteins was detected. MTBP is reminiscent of Sld7 in its binding to Treslin/TICRR/Sld3. This binding appears essential for replication as MTBP nonbinding Treslin/TICRR mutants did not facilitate replication. These functional similarities of MTBP and Sld7, and similarities in protein sequence and structure of the C termini [29] led to the hypothesis that MTBP performs Sld7-like functions in metazoans. However, no statistically significant evidence for orthology between MTBP and Sld7 has been provided.

We here employed various approaches to search for remote homologies in the MTBP and Sld7 proteins. These revealed MTBP to possess two Sld7-homologous regions in its N and C termini, and a metazoa-specific region separating these two homology domains. We show that the Sld7-homologous domains are required for proper replication origin firing in human cells. We thus incontrovertibly demonstrate orthology between MTBP and Sld7. This fills the last gap in the list of metazoan core origin firing factors, establishing a universal framework of eukaryotic replication initiation. Despite this conservation, metazoa have also evolved specific initiation processes, because the metazoa-specific middle domain of MTBP proved to be required for proper DNA replication. This domain apparently harbours more than one activity important for replication. Cyclin-dependent kinase 8/19–cyclinC (Cdk8/19-cyclin C), a protein that was not previously implicated in DNA replication, with roles in controlling transcription [30], binds the metazoa-specific MTBP domain. This interaction was required for complete genome replication and, consequently, for normal chromosome segregation. We hypothesise that the metazoa-specific binding of Cdk8/19-cyclin C to MTBP helps integrate the conserved initiation principles into the special requirements of the more complex metazoan cells to achieve well-regulated origin firing to guarantee genome stability.

## Results

### Both termini of MTBP possess Sld7-homologous domains

Human MTBP (hMTBP) is surprisingly devoid of known domain homologues. To identify its domain architecture, we initiated an exhaustive computational sequence analysis. We identified three domains that are conserved in MTBP orthologues across most of the animal kingdom.

**Fig 1:**
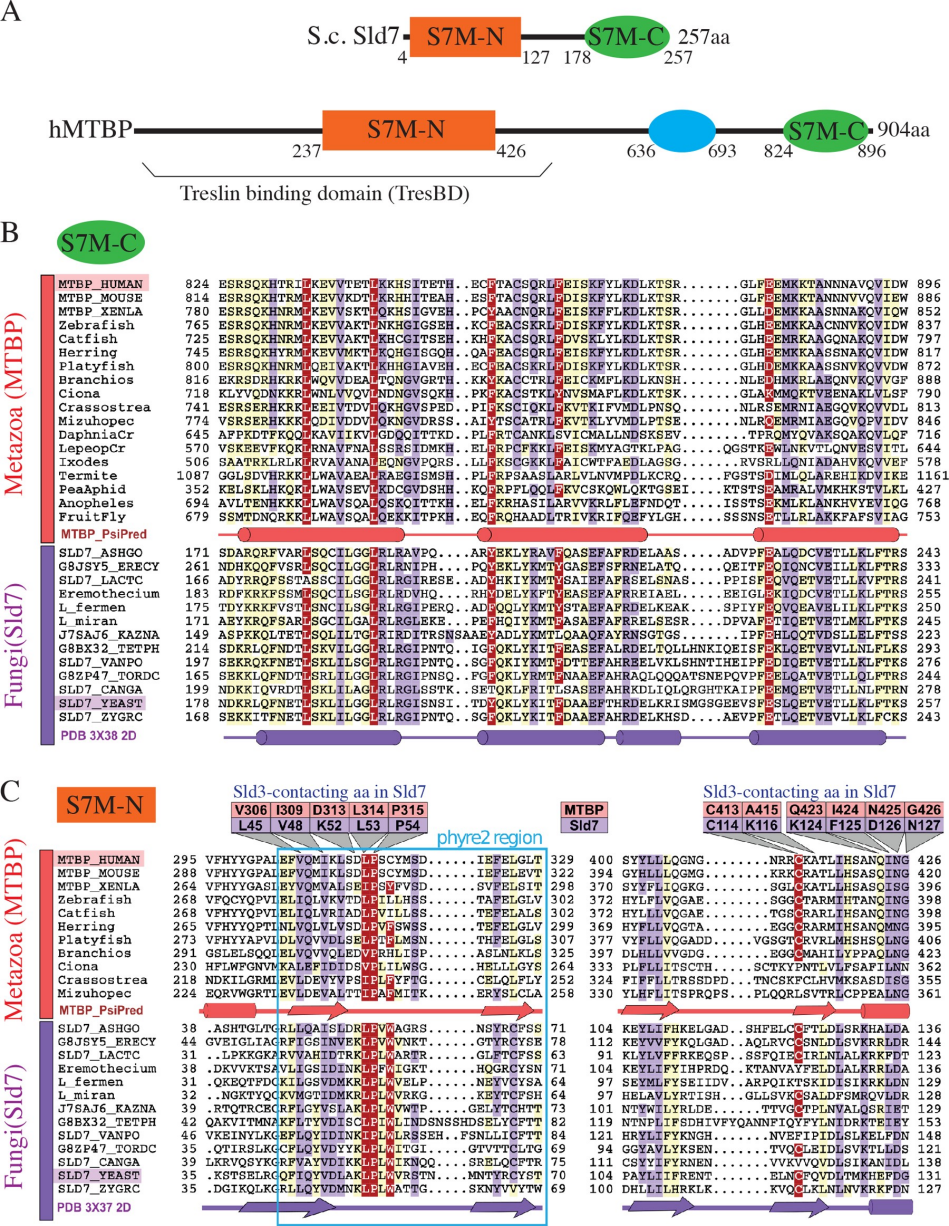
hMTBP and yeast Sld7 share two homologous domains, Sld7-MTBP-N and -C (S7M-N and -C). (A) Domain architecture of hMTBP and S.c. Sld7. S7M-N, -C, conserved domains; blue oval, metazoa-specific domain. (B,C) Multiple sequence alignments of (B) S7M-C and (C) S7M-N domains. Sequences from representative metazoa (red) or fungi (purple) presented with Belvu using a colouring scheme indicating the average BLOSUM62 scores (that correlate with amino acid conservation): red (>2.5), violet (between 2.5 and 0.8), and light yellow (between 0.8 and 0.3). Predicted or known secondary structures are indicated: cylinders, α-helices; arrows, β-strands. Amino acids discussed in the main text are indicated with hMTBP or yeast Sld7 numbering (panel C). Blue box (panel C) indicates the phyre2 region. aa, amino acid; BLOSUM62, blocks substitution matrix 62; hMTBP, human MTBP; MTBP, Mdm2 binding protein; phyre2, protein homology/analogy recognition engine V 2.0; S.c., *Saccharomyces cerevisiae*; Sld7, synthetic lethal with Dpb11; S7M-C, Sld7-MTBP C-terminal domain; S7M-N, Sld7-MTBP N-terminal domain.

Two of these domains proved conserved in yeast Sld7 (Fig 1A). For this we employed iterative profile-based sequence similarity searches [31] of the UniRef50 database [32]. Focusing first on the most C-terminal of these regions, we found that its sequences are statistically significantly similar to the C terminus of *Saccharomyces cerevisiae* Sld7 of known tertiary structure (protein data bank [PDB] identifier, 3×38) [18] (*E*-value = 0.012; probability = 94.1%) (Fig 1B).

This step employed HHpred searches against the PDB70 profile database [33] and a C-terminal conserved region, amino acids 824–896 of hMTBP, as input. Consistent with these C-terminal domains of MTBP and Sld7 being homologous, secondary structure predictions of a three-helix bundle for the former agree with the published Sld7 crystal structure [18, 34]. Thus, the C-terminal domains of MTBP and Sld7 have evolved from a common ancestral sequence. We call them the Sld7-MTBP C-terminal domain (S7M-C). Our findings provide the statistical evidence required to support a recent proposal of MTBP-Sld7 C-terminal domain homology [29].

Two complementary approaches then provided evidence for MTBP and Sld7 sharing an N-terminal homologous domain. The first approach queried the protein homology/analogy recognition engine V 2.0 (phyre2) server with MTBP sequences and returned low confidence alignments with yeast Sld7 (S1A Fig) [18]. We term these amino acids the MTBP-phyre2 and Sld7-phyre2 region. Six amino acids in the Sld7-phyre2 region directly contact Sld3 to form Sld7-Sld3 dimers (L45, V48, 52-KLPL-55 of *Zygosaccharomyces rouxii* Sld7; S1A Fig, blue asterisks; S2 Fig) [18], and four of them are conserved in MTBP (V306, I309, L314, P315) with respect to their chemical properties. These MTBP amino acids are among the most highly conserved residues in this region across animals (S1B Fig).

**Fig 2:**
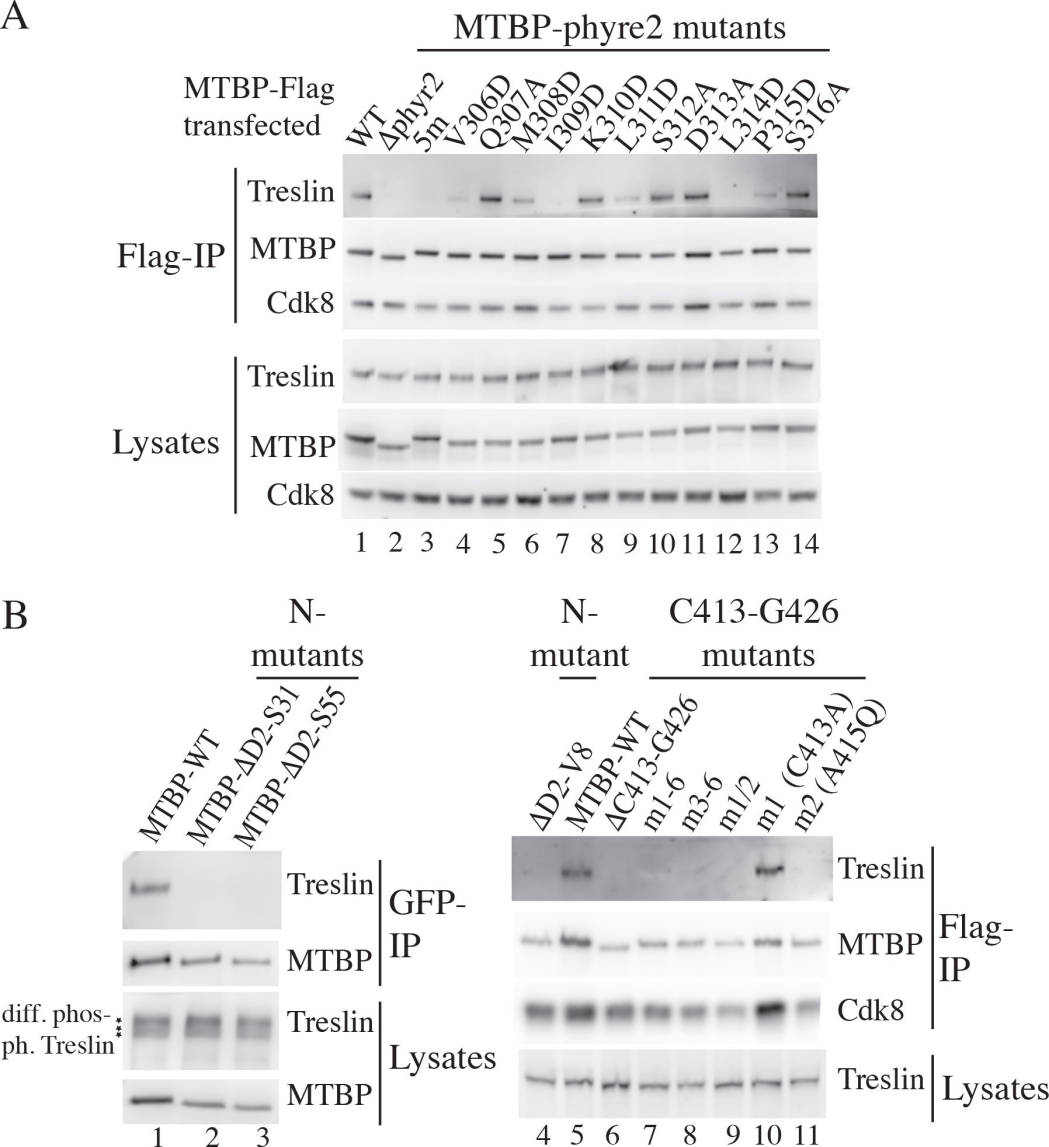
The N termini of MTBP and Sld7 share a function, interaction with Treslin/TICRR/Sld3. (A) C-terminally 3×Flag-tagged MTBP-WT, deletion (Δ), or point mutants (m) in the MTBP-phyre2 region were transiently transfected into 293T cells before analysis by anti-Flag IP and immunoblotting using antibodies against MTBP (12H7) and Treslin/TICRR (30E7). Δphyr2, amino acids V295-T329 deleted; 5m, amino acid exchanges V306D, I309D, D313A, L314D, P315D. (B) Experiment as in (A) except lanes 1–3: C-terminally Flag-GFP-tagged MTBP was immunoprecipitated using anti-GFP nanobodies. *, different Treslin/TICRR phosphorylation forms visible in some immunoblots; point mutations: m1–6, C413A, A415Q, Q423A, I424Q, N425A, G426Q; m3–6, Q423A, I424Q, N425A, G426Q; m1/2, C413A, A415Q; m1, C413A, m2, A415Q. Cdk8, cyclin-dependent kinase 8; GFP, green fluorescent protein; IP, immunoprecipitation; m, point mutant; MTBP, Mdm2 binding protein; Sld7, Synthetic lethal with Dpb11; TICRR, TopBP1-interacting checkpoint and replication regulator; WT, wild-type.

We tested next if these amino acids in the MTBP-phyre2 region are important for binding to Treslin/TICRR. We deleted the phyre2 region (amino acids V295-T329) of hMTBP (MTBP-Δphyr2) and tested its interaction with endogenous Treslin/TICRR in cell lysates after transient transfection of MTBP-Flag into 293T cells. Flag immunoprecipitation (IP) (see Table 1 for all antibodies used) of wild-type (WT) MTBP-Flag (MTBP-WT), but not MTBP-Δphyr2, co-purified Treslin/TICRR (Fig 2A, lanes 1 and 2). A quintuple point mutant (MTBP-5m) exchanging the MTBP-phyre2 region amino acids V306, I309, D313, L314, and P315 against alanine (D313) or aspartate (all others) also showed no detectable binding to Treslin/TICRR (lane 3). These five residues map to Sld3-contacting amino acids in Sld7 (Figs 1C and S2). MTBP-Δphyr2 and MTBP-5m were specifically defective in binding to Treslin/TICRR but bound Cdk8, a new MTBP interactor, whose function in replication we discuss below, as well as MTBP-WT, suggesting that the mutants are not misfolded. To assess further the folding quality of the MTBP-5m protein, we tested its migration behaviour in gel filtrations. We found that MTBP-WT and MTBP-5m eluted indistinguishably from each other as sharp peaks and did not form aggregates (S3 Fig). MTBP-5m localises predominantly to the nucleus, like MTBP-WT (S4 Fig).

**Table 1:** Specification of the antibodies used.

Name	Protein/Peptide	Species/Source	Distributor
anti-MTBP clone 4H9	hMTBP, amino acids 102–513	rat	self-made [28]
anti-MTBP clone 12H7	hMTBP, amino acids 1–284	rat	self-made [28]
anti-MTBP (83)	hMTBP; amino acids 1–284	rabbit	self-made
anti-MTBP (85)	hMTBP; amino acids 102–513	rabbit	self-made
anti-Treslin clone 30E7	human Treslin/TICRR	mouse	self-made
anti Treslin DB148	human Treslin/TICRR; amino acids 1566–1910	rabbit	self-made [26]
anti Treslin DB153	human Treslin/TICRR, amino acids 970–1400	rabbit	self-made [26]
anti-BrdU-FITC set	BrdU	mouse	BD 556028
anti-FLAG M2 Monoclonal antibody	FLAG	mouse	Sigma-Aldrich, F1804-200UG
anti-TopBP1 antibody (DB4)	human TopBP1 amino acids 1–360	rabbit	self-made [26]
anti-TopBP1 antibody (DB7)	human TopBP1 amino acids 430–800	rabbit	self-made [26]
anti-cyclin C	human cyclin C	rabbit	Bethyl/Biomol (A301-989A)
anti-Cdk19	human Cdk19	rabbit	Sigma (HPA007053)
anti-Cdk8	human Cdk8	goat	Santa Cruz (sc-1521)
anti-GFP nanobodies	GFP	recombinant purified from *Escherichia coli*	generous gift from Kirill Alexandrov
anti-GFP (JL-8)	GFP	mouse	Takara (632381)
anti-Med12	Med12	goat	Santa Cruz (sc-5374)
anti-Med13	Med13	goat	Santa Cruz (sc-12013)
anti-mouse Alexa 488	IgG	rabbit	Invitrogen, A11059
anti-α tubulin	α tubulin	mouse	Sigma T5168
anti-Cdc45 clone 3G10	human Cdc45	rat	Kind gift of H. Pospiech und F. Grosse
anti-Mcm5	human Mcm5	rabbit	Abcam, ab17967
anti-Sld5	human Sld5	rabbit	Abcam, ab139683

Abbreviations: BD, Becton Dickinson; BrdU, 5-bromodeoxyuridine; Cdc, cell division cycle; Cdk, cell cycle dependent kinase; GFP, green fluorescent protein; hMTBP, human MTBP; IgG, immunoglobulin; Mcm, minichromosome maintenance; Med, mediator of transcription; MTBP, Mdm2 binding protein; Sld, synthetic lethal with Dpb11; TICRR, TopBP1 interacting checkpoint and replication regulator; TopBP1, Topoisomerase II binding protein.

We realised that in some gels Treslin/TICRR in samples from cell lysates formed multiple bands representing different phosphorylation forms, whereas MTBP-bound Treslin/TICRR usually showed only one band (Fig 2B left panel). To test if MTBP-bound Treslin/TICRR is phosphorylated we ran lambda phosphatase (PPase)-treated and untreated lysates side by side with MTBP immunoprecipitates on an SDS polyacrylamide gel to compare gel mobility of Treslin/TICRR. MTBP-bound Treslin/TICRR showed a PPase-dependent mobility shift (S5 Fig).

To fine-map the interaction between MTBP and Treslin/TICRR, we then mutated amino acids V306 to S316 in the MTBP-phyre2 region individually. MTBP-Flag IP showed that four of the five amino acids aligning with Sld3-interacting amino acids, V306, I309, L314, and P315 (S2 Fig), contribute to Treslin/TICRR binding (Fig 2A, lanes 4, 7, 12, 13). Mutating other amino acids that were not aligned with Sld3-interacting Sld7 amino acids had variable effects. Mutating the most conserved, L311 (lane 9), showed a considerable reduction of Treslin/TICRR binding. Mutating the moderately conserved M308 (lane 6) had mild effects, whereas mutating the moderately conserved Q307 (lane 5) and the weakly conserved K310 and S312 (lanes 8 and 10) did not alter Treslin/TICRR interaction. All mutants bound Cdk8 normally. These data show that the phyre2 regions of Sld7 and MTBP have a conserved function, the binding to Sld3/Treslin/TICRR.

To identify homology between MTBP and Sld7 N termini, we then used the newly identified MTBP-phyre2 region as an anchor point for similarity searches based on amino acid sequences and secondary structures. This revealed extensive sequence similarities between amino acids V295 and G426 in the N terminus of MTBP and the full length of the N-terminal domain of Sld7 (Figs 1C and S2A). These similarities are sufficient to predict homology because their alignment score greatly exceeds those expected from alignments with random sequences (*E* = 3.0 × 10^−5^). We term these domains in Sld7 and MTBP Sld7-MTBP N-terminal (S7M-N) domains. Independent evolution of shared domain architectures is rare [35] and highly unlikely for functionally similar proteins. Our evidence thus strongly indicates that metazoan MTBP and fungal Sld7 are orthologues.

Regions outside MTBP-phyre2 are also important for interaction with Treslin/TICRR. Itou and colleagues showed that the S7M-N domain of Sld7 contains a second cluster of Sld3-contacting amino acids (Fig 1C) [18]. The corresponding positions in the S7M-N domain of hMTBP are amino acids C413–G426, and were also required for interaction with Treslin/TICRR (Fig 2B lanes 5–11). MTBP-Q423-G426 (mutated in MTBP-m3–6) and C413 (MTBP-m1) map to positions in Sld7 that directly contact Sld3 (S2 Fig). A415 (MTBP-m2) probably affects the stability of its β-strand (S2 Fig). Finally, deleting amino acids D2 to S55, D2 to S31 or D2 to V8 from the N terminus also resulted in MTBP mutants that failed to bind Treslin/TICRR (Fig 2B lanes 1–5). MTBP-ΔD2-V8 and mutants of the region between amino acids C413 and G426 do not show signs of protein misfolding. Their Cdk8 binding ability (Fig 2B) and migration behaviour in gel filtrations were indistinguishable from MTBP-WT (S3 Fig). MTBPm1–6 localises predominantly to the nucleus, like MTBP-WT (S4 Fig). We conclude that various regions in the N terminus of MTBP contribute to Treslin/TICRR binding, and termed it Treslin/TICRR binding domain (TresBD).

### The S7M-N and -C domains function in DNA replication

**Fig 3:**
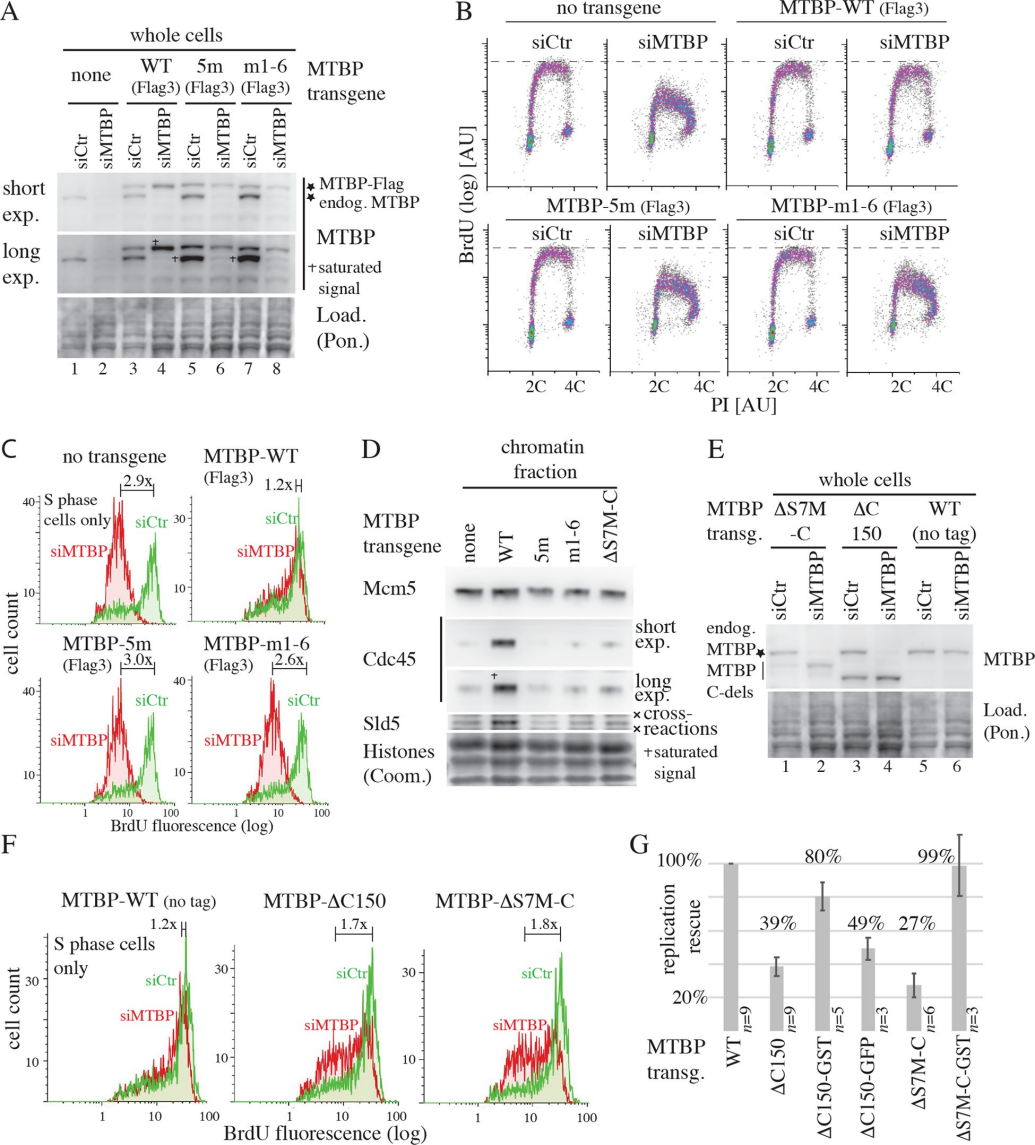
The S7M-N and -C domains are required for DNA replication in human cells. (A) HeLa Flip-In T-Rex cell lines carrying no transgene, siRNA-resistant MTBP-WT-3×Flag, deletion (Δ), or point mutants (m) were treated with siCtr or siMTBP and doxycycline. Whole cell lysates were immunoblotted using anti-MTBP antibody (12H7). Ponceau (Pon.) staining controlled loading (Load.). Short exp., short exposure; long exp., long exposure; 5m and m1–6, see Fig 2 for nomenclature. (B) Flow cytometry density plots of cells treated as in (A), stained by BrdU pulse labelling and PI. Relative DNA content before (2C) and after (4C) replication. (C) Histogram overlays of BrdU fluorescence of S phase populations of cells treated and analysed as in (B). Numbers indicate times reduction of BrdU fluorescence upon siMTBP treatment. (D) The indicated Hela Flp-In cell lines were siMTBP treated as in (A). Chromatin fractions were tested by immunoblotting using antibodies against Cdc45, Sld5, Mcm5. Coomassie (Coom.) staining of histones controlled equal chromatin preparation and loading. (E, F) Experiment as in (A) and (C). MTBP mutants: ΔS7M-C, C-terminal 81 amino acids deleted; ΔC150, C-terminal 150 amino acids deleted. (G) Quantification of multiple (*n*) independent experiments with the indicated cell lines as in (A) and (B). Percent replication rescue represent normalisations to MTBP-WT (100%) and no-transgene cells (0%); error bars, SEM. AU, arbitrary unit; BrdU, bromodeoxyuridine; Cdc45, cell division cycle 45; Coom., Coomassie; Load., Loading; log, logarithmic scale; long exp., long exposure; m, point mutant; Mcm5, minichromosome maintenance 5; MTBP, Mdm2 binding protein; PI, propidium iodide; Pon., Ponceau; Short exp., short exposure; siRNA, small interfering RNA; siCtr, control siRNA; siMTBP, MTBP siRNA; Sld5, Synthetic lethal with Dpb11 5; S7M-N, Sld7-MTBP N-terminal domain; S7M-C, Sld7-MTBP C-terminal domain; WT, wild-type.

We next investigated if the two Sld7-like domains of MTBP, S7M-N and S7M-C, are important for DNA replication. We used an established RNA interference (RNAi) system [28] (see Table 2 for all small interfering RNAs [siRNAs] used) to replace MTBP with siRNA-resistant MTBP-WT or mutant MTBP transgenes (Fig 3A). Relative replication speed was then determined by 5-bromodeoxyuridine (BrdU) labelling and flow cytometry (Fig 3B and 3C, gating strategy for all flow cytometry experiments, including raw data for quantification in S1 Data). In control cells expressing no transgene MTBP-RNAi (siMTBP) reduced the BrdU signal 2.9-fold compared with control RNAi (siCtr) (Fig 3C and S1 Data), indicating effective suppression of replication. siRNA-resistant MTBP-WT rescued replication in siMTBP-treated cells almost completely (1.2× reduction).

**Table 2:** Specification of the siRNAs used.

Target	Sequence	Distributor
MTBP1	GAGAGAAACAGUUAGCUAA	J-013953-06-0050 Dharmacon
MTBP2	UCACAUUGUUGGAUGCUAA	J-013953-08-0050 Dharmacon
Control/GL2	CGUACGCGGAAUACUUCGAUU	CTM-134302 Dharmacon
Med12-1		D-009092-02 Dharmacon
Med12-2		D-009092-04 Dharmacon
Treslin/TICRR	GAACAAAGGTTATCACAAA	Dharmacon; custom-made
CycC-1		D-003209-06 Dharmacon
CycC-2		D-003209-21 Dharmacon

Abbreviations: CycC, cyclin C; GL2, siRNA against firefly luciferase gene; Med12, mediator of transcription 12; MTBP, Mdm2 binding protein; TICRR, TopBP1 binding checkpoint and replication regulator.

In contrast, Treslin/TICRR nonbinding S7M-N mutants, MTBP-5m (phyre2 region) and MTBP-m1–6 (amino acid C413–G426 region), did not rescue replication. These mutants’ BrdU–propidium iodide (PI) profiles were indistinguishable from siMTBP-treated control cells. BrdU signals were reduced 3.0-fold (MTBP-5m) and 2.6-fold (MTBP-m1–6). We sought to use minimally invasive single point mutations to confirm these conclusions that were drawn from the MTBP-5m and m1–6 combination mutants. The three non-Treslin/TICRR-binding MTBP mutants I309D, L314D (both mutated in 5m) and A415Q (mutated in m1–6) did not support replication over background, whereas cells expressing the Treslin/TICRR-binding proficient D313A replicated normally (S6 Fig and S1 Data).

To test which step of DNA replication was defective in Treslin/TICRR nonbinding MTBP mutants, we tested if origin licensing and origin firing occurred in cells expressing these mutants. Immunoblotting of chromatin fractions indicated normal licensing, as judged by Mcm5 (pre-RC subunit) signals (Figs 3D and S7 and S1 Data). In contrast, Cdc45 and Sld5 (GINS subunit) antibodies indicated that CMGs did not form in the presence of the MTBP mutants, unlike with MTBP-WT. Together, we conclude that the Treslin/TICRR-binding S7M-N region is essential for MTBP function in replication at the origin firing step.

These conclusions are consistent with earlier results showing that Treslin/TICRR mutants that do not bind MTBP are replication incompetent [28]. We reported before that binding of Treslin/TICRR to MTBP is required for normal cellular levels of MTBP [28] (S8E and S8F Fig and S1 Data). Consistently, the Treslin/TICRR binding-deficient MTBP-5m and m1–6 transgenes, but not other mutants that can bind Treslin/TICRR, expressed to lower levels than MTBP-WT (Figs 3A and S8A and S6A and S1 Data). Cycloheximide shut-off experiments with MTBP-5m and m1–6 confirmed that these degrade faster than MTBP-WT (S8B Fig). Lower MTBP levels cannot be the sole reason for the lack of replication in MTBP-5m and m1–6 cells because partial siMTBP-mediated knock-down experiments showed that strong replication defects required suppression of MTBP signals to less than approximately 15%, whereas the 5m and m1–6 mutants have approximately 40% and 35% of MTBP signals left compared with endogenous and transgenic MTBP-WT (S8A, S8C and S8D Fig and S1 Data). We then tested if elevating the expression levels of the MTBP-5m and m1–6 to MTBP-WT levels rescues their replication deficiency. To this end we selected stable Hela-Kyoto cell clones generated by random transgene integration into the genome that expressed MTBP-5m, m1–6, or WT at comparable levels. Replication analysis showed very low if any replication activity of the MTBP mutants (S9 Fig and S1 Data), indicating Treslin/TICRR binding to MTBP has essential roles in replication distinct from or in addition to stabilising MTBP levels.

The S7M-C domain is also important for replication. Two MTBP mutants lacking the C-terminal 150 (MTBP-ΔC150) or 81 amino acids (MTBP-ΔS7M-C) showed defects in rescuing replication. BrdU signals were reduced by 1.7-fold in MTBP-ΔC150 and 1.8-fold in MTBP-ΔS7M–C (Fig 3F and S1 Data). Because these replication defects were milder compared with the Treslin/TICRR-binding mutants, we quantified replication of these mutants more thoroughly. Averaging multiple independent experiments confirmed that both mutant cell lines replicated significantly less than MTBP-WT–expressing lines (39% and 27% of WT) (Fig 3G and S1 Data). Both mutant proteins bound Treslin/TICRR normally (S10A Fig). Moreover, chromatin immunoblots showed that, whereas licensing occurred normally in cells expressing MTBP-ΔS7M-C, origin firing (CMG formation) was impaired, albeit not as much as in Treslin/TICRR-nonbinding MTBP mutants (Figs 3D and S7 Fig and S1 Data). Together, we conclude that the S7M-C domain is required for MTBP function in replication origin firing. The S7M-C function is distinct from Treslin/TICRR binding, consistent with a recent report [29]. The Sld7-Sld3 crystal structure suggested that the S7M-C domain of Sld7 mediates Sld7 dimerisation [18]. The relevance of Sld7 dimerisation for replication in yeast cells was not tested. If the S7M-C domain of MTBP mediates replication by homodimerisation, fusing a homodimerisation domain to MTBP that lacks the S7M-C domain should rescue the capability of this MTBP mutant to induce replication. We attached MTBP-ΔC150 and MTBP-ΔS7M-C with their C termini to the homodimerising glutathione S transferase (GST) tag. Cells expressing MTBP-ΔC150-GST and MTBP-ΔS7M-C-GST transgenes replicated to near WT levels, 80% and 99%, respectively, whereas fusing the nondimerising green fluorescent protein (GFP) tag had little effect (Figs 3G and S10B and S1 Data). The slightly better replication by the GFP fusion (49%) over MTBP-ΔC150 (39%) might stem from its higher expression level. The most parsimonious explanation of the replication rescue by fusing GST is that GST replaces a dimerisation activity of the C terminus, and there is no other essential replication function of the C terminus. However, alternative, more complicated scenarios cannot be excluded.

### MTBP requires the metazoa-specific region to support normal genome replication

**Fig 4:**
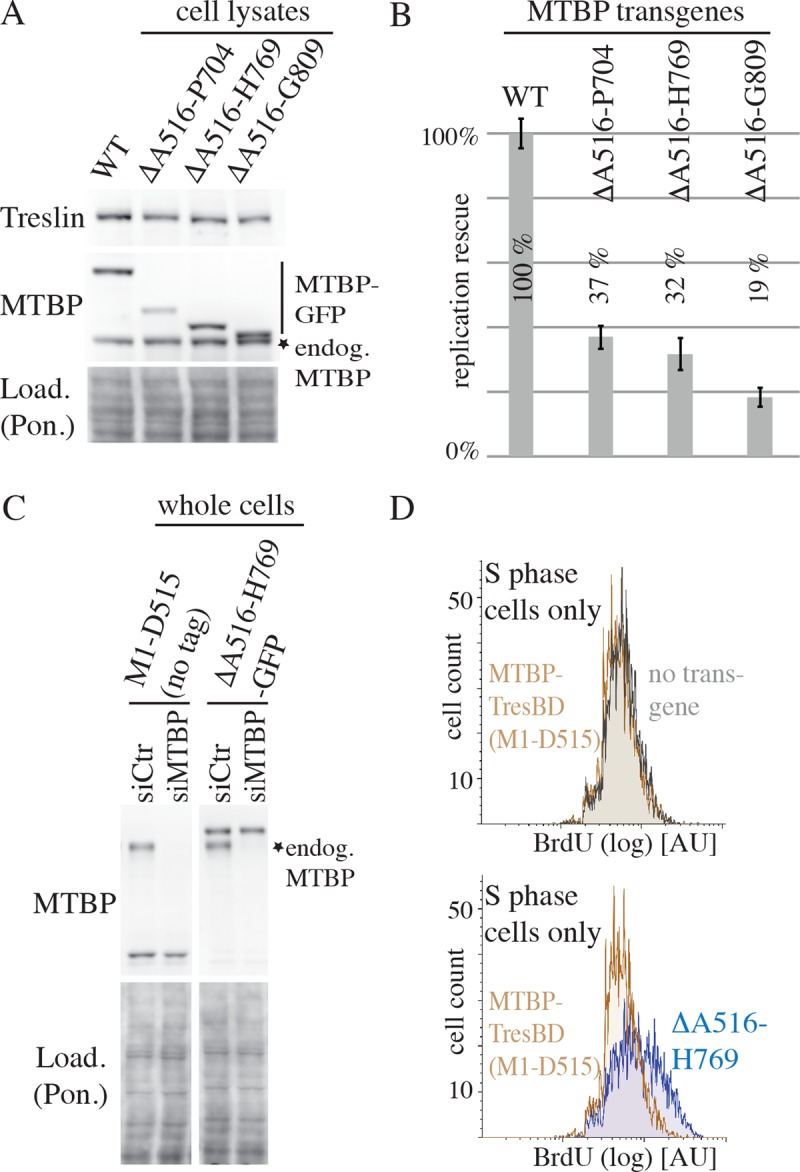
The metazoa-specific region of MTBP is required for WT levels of DNA replication. (A) Lysates of HeLa Flip-In T-Rex cells expressing siMTBP-resistant MTBP-WT-3×Flag-TEV2-GFP, MTBP-Δ516–704–3×Flag-TEV2-GFP (lacking amino acids A516–P704), MTBP-Δ516–769–3×Flag-TEV2-GFP-NLS, or MTBP-Δ516–809–3×Flag-TEV2-GFP-NLS were analysed by immunoblotting using anti-MTBP (4H9), anti-Treslin (148), and Ponceau (Pon.) staining. Error bars: SEM. (B) Cell lines of (A) were siMTBP- or siCtr-treated and analysed for replication rescue as described in Fig 3G. Averages of two (MTBP-Δ516–704–3×Flag-GFP) or four (all other lines) independent experiments are shown. (C) Whole cell lysates of HeLa Flip-In T-Rex cells expressing siMTBP-resistant MTBP-TresBD (amino acids 1–515) or MTBP-Δ516–769–3×Flag-TEV2-GFP-NLS were siCtr- or siMTBP-treated and analysed by immunoblotting as in Fig 3A. Crops of the same immunoblot exposure are shown. (D) S phase populations of cells described in (C) were analysed by BrdU-flow cytometry as in Fig 3C. AU, arbitrary units; BrdU, 5-bromodeoxyuridine; GFP, green fluorescent protein; Load., loading; MTBP, Mdm2 binding protein; Pon., Ponceau; siCtr, control RNAi; siMTBP, MTBP-RNAi; TresBD, Treslin/TICRR binding domain; WT, wild-type.

We hypothesised that the Sld7-homologous domains of MTBP confer the core replication activities common across eukaryotes, whereas the metazoa-specific MTBP middle region may mediate metazoa-specific roles in replication, or may have nonreplicative functions. To test the relevance of the MTBP middle domain for replication, we sought to delete the whole middle domain but retain the activities of the S7M termini. The smallest TresBD fragment of MTBP that was biochemically well behaved (was soluble and showed no unspecific binding to control pull-down beads) and retained full Treslin/TICRR binding activity contained amino acids M1 to Y513 (S11A Fig). We fused to it amino acids L705 to K904, Y770 to K904, or Q810 to K904 (Fig 4A). We call these mutants metazoan Sld7s (mSld7s) because they contain little more than the Sld7-equivalent MTBP termini. All three mSld7 versions supported replication poorly compared with MTBP-WT (Fig 4B and S1 Data) but better than cells carrying no transgene, cells expressing the TresBD (amino acids M1–D515) (Fig 4C and 4D and S1 Data), and cells that lack TresBD activity (Fig 3A–3C and S1 Data).

This shows that the Sld7-equivalent termini cooperate in mSld7s to mediate replication, albeit to sub-WT levels. Because MTBP-ΔA516-H769 and ΔA516-G809 lack an nuclear localisation sequence (NLS) around amino acid D740, a simian virus 40 (SV40)–NLS had been fused to the C terminus of the C-terminal GFP tag. All mSld7 versions bound Treslin/TICRR. ΔA516–P704 and ΔA516–H769, but not ΔA516–G809, showed some reduction of Treslin/TICRR association compared to WT (S11B Fig). Because the C-terminal 388 amino acids of MTBP (amino acids A516–K904) are dispensable for Treslin/TICRR binding (S11A Fig), this indicates that the artificial domain boundaries in these two mutants may affect folding of the N-terminal TresBD to some degree. The relatively weak Treslin/TICRR binding of MTBP-ΔA516–P704 is probably the reason for the slightly lower expression of this transgene. We do not think, however, that lower Treslin/TICRR binding capacity and lower MTBP protein levels are critical for the low replication in these mutant cells, because the Treslin/TICRR binding capacities of the mSld7 versions do not correlate with their replication-promoting activities (Figs 4B and S11B). We conclude that the metazoa-specific middle domain of MTBP and the Sld7-homologous domains cooperate to support DNA replication in human cells.

### Cdk8/19-cyclin C is a novel interactor of the MTBP-Treslin/TICRR-TopBP1 core initiation complex

**Fig 5:**
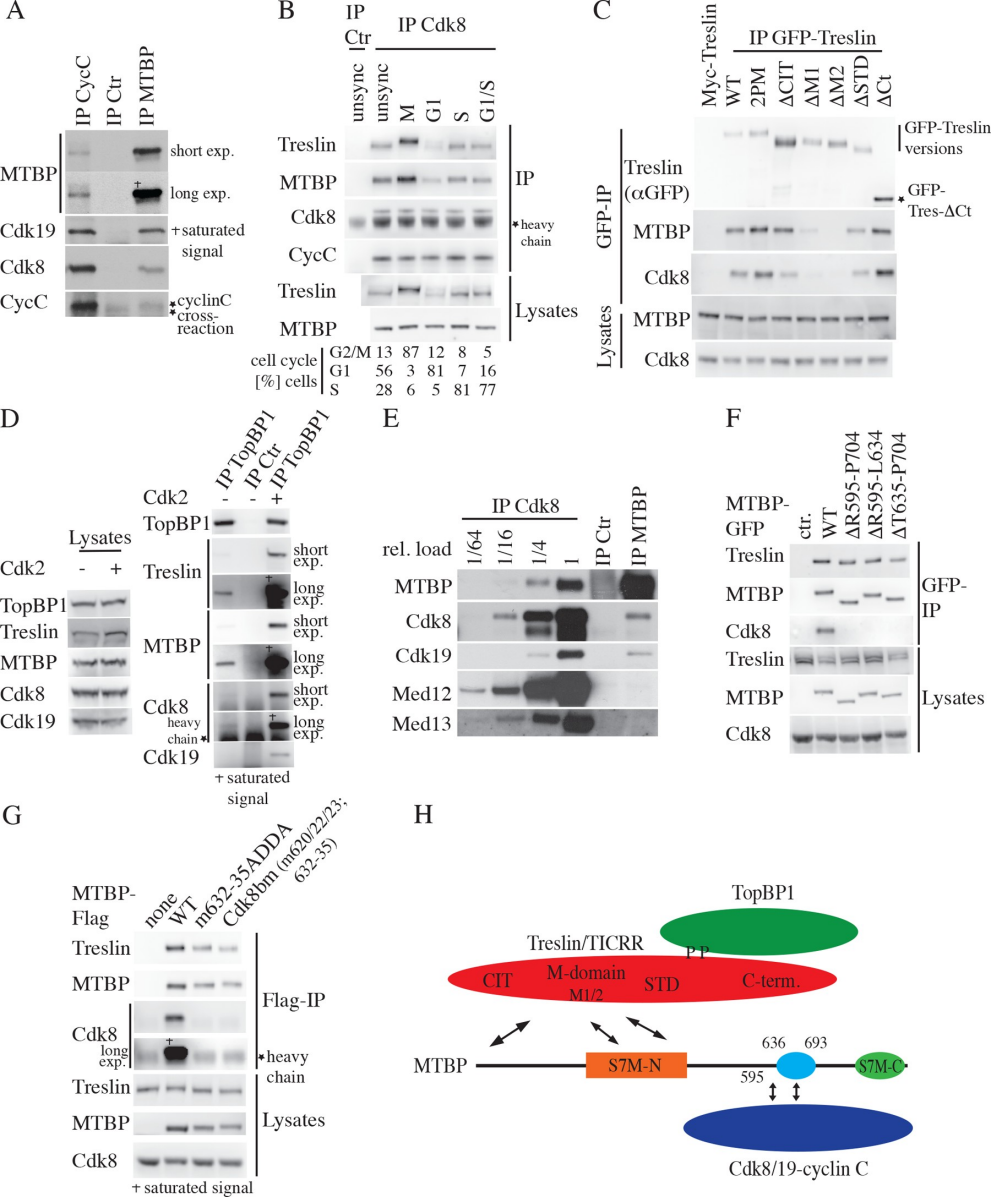
Cdk8/19-cyclin C forms a protein complex with Treslin/TICRR-MTBP-TopBP1 that is independent from mediator of transcription subunits. (A) Native lysates of HeLa Flip-In T-Rex cells were immunoprecipitated using IgG, anti-MTBP (83), or anti-cyclin C antibodies. Immunoblots were detected using anti-MTBP (85), anti-cyclin C, anti-Cdk19, and anti-Cdk8. (B) Native cell lysates of unsynchronised cells or cells in mitosis (M) (nocodazole arrest), G1 phase (4h nocodazole release), G1/S (thymidine arrest), or S (3h thymidine release) were analysed by IP using anti-Cdk8 or control IgG and immunoblotting with anti-Cdk8, anti-MTBP (4H9), and anti-Treslin/TICRR (30E7) antibodies. Cell cycle distributions were determined by PI flow cytometry. (C) 293T cells transiently transfected with GFP-Flag-Treslin-WT or the mutants Treslin-2PM (Fig 5H) (TopBP1 binding deficient), -ΔCIT, -ΔM1/2, -ΔSTD, -ΔCt were analysed by anti-GFP IP and immunoblotting using anti-GFP, anti-MTBP (4H9), and anti-Cdk8 antibodies. (D) Native lysates of 293T cells were treated with Cdk2-cyclin A or left untreated before IP with anti-TopBP1 antibodies and immunoblotting using anti-TopBP1, anti-Treslin (30E7), anti-Cdk8, and anti-Cdk19 antibodies. (E) Native lysates of HeLa-Kyoto cells were immunoprecipitated with control IgG, anti-MTBP (83), or anti-Cdk8 antibodies. Different relative amounts (1 to 1/64) were analysed by immunoblotting with anti-MTBP (4H9), anti-Cdk8, anti-Cdk19, anti-Med12, and anti-Med13 antibodies. Stronger bands were in saturated signal range. (F) Native lysates of HeLa Flip-In T-Rex cells expressing MTBP-WT-Flag3-GFP or mutants lacking the indicated amino acids were analysed by anti-GFP IP and immunoblotting using anti-MTBP (4H9), anti-Treslin (30E7), and anti-Cdk8 antibodies. (G) Flag immunoprecipitates and lysates of HeLa Flip-In T-Rex cells expressing MTBP-WT-Flag3 or indicated mutants were analysed by immunoblotting using anti-MTBP (4H9), anti-Cdk8, and anti-Treslin (30E7) antibodies. (H) Schematic of the TopBP1-Treslin/TICRR-MTBP-Cdk8/19-cyclin C protein complex. The Treslin domains (see Materials and methods for definition) CIT, Middle (M) domain divided into M1 and M2, STD, and C-ter are indicated. ΔCIT, conserved in Treslins domain deleted; ΔCt, C terminus deleted; ΔM1/2, nonoverlapping M-domain deletion mutants, ΔSTD, Sld3/Treslin domain deleted; Cdk8/19, cyclin-dependent kinase 8/19; CIT, conserved in Treslins; C-ter, C terminus; Ctr, control; CycC, cyclin C; exp., exposure; GFP, green fluorescent protein; heavy chain, heavy antibody chain; IgG, immunoglobulin; IP, immunoprecipitation; MTBP, Mdm2 binding protein; Myc, myelocytomegalovirus proto oncogene; PI, propidium iodide; Sld3, synthetic lethal with Dpb11; STD, Sld3/Treslin domain; S7M-C, Sld7-MTBP C-terminal domain; S7M-N, Sld7-MTBP N-terminal domain; TICRR, TopBP1 interacting checkpoint and replication regulator; TopBP1, Topoisomerase II binding protein; unsync, unsynchronised; WT, wild-type.

We found, using purification of MTBP-Flag-GFP and mass spectrometry, that the Cdk8 and Cdk19 kinases and their activating subunit cyclin C co-purify with MTBP. Cdk8 and Cdk19 show extremely high sequence identity. In our experiments they behaved identically, and we do not functionally distinguish between them. Endogenous MTBP co-purified Cdk8, cyclin C, and Cdk19, and anti-cyclin C antibodies precipitated Cdk8, Cdk19, and MTBP (Fig 5A). The interaction occurred in lysates from asynchronous cells and from cells synchronised in G1, S, or mitosis (Fig 5B). The slightly lower MTBP amounts in G1 cell lysates suggested that the interaction with Cdk8/19-cyclin C might require cell cycle CDK kinase activity, but adding Cdk2-Cyclin A to G1 lysates did not promote the interaction. Treslin/TICRR also co-purified with anti-Cdk8 antibodies from all cell cycle stages (Fig 5B).

Moreover, transiently transfected Treslin/TICRR-WT and mutants of the Treslin/TICRR termini, the Sld3/Treslin domain (STD), and the TopBP1 interaction domain coimmunoprecipitated Cdk8/19-cyclin C (Fig 5C). In contrast, two deletion mutants of the Treslin/TICRR M domain that are partially (ΔM1) or strongly (ΔM2) deficient in MTBP interaction showed proportional defects in Cdk8 binding. These interaction studies suggested that MTBP, CDK8/19-cyclin C, and Treslin/TICRR form a protein complex, with MTBP bridging Treslin/TICRR and the Cdk8 kinase (Fig 5H). Corroborating this interpretation, a C-terminal MTBP-A516–K904 fragment, but not the TresBD fragment, interacted with Cdk8/19-cyclin C (S12B(i) Fig), placing the binding site for the kinase between MTBP amino acids A516 and K904.

TopBP1 is also part of the Treslin/TICRR-MTBP-Cdk8/19-cyclin C complex. IP of endogenous TopBP1 from cell lysates co-purified Treslin/TICRR, MTBP, and a faint but specific signal for Cdk8 (Fig 5D). Adding Cdk2-cyclin A to the lysate to enhance the binding of Treslin/TICRR to the N-terminal triple breast cancer type 1 susceptibility protein C terminal repeat (BRCT) repeat domain of TopBP1 [26] increased the signals for Treslin/TICRR, MTBP, Cdk8, Cdk19, and cyclin C, indicating that TopBP1 indirectly binds MTBP-Cdk8/19-cyclin C via Treslin/TICRR (Fig 5H).

Cdk8-cyclin C forms the kinase module of the mediator of transcription together with mediator of transcription subunits 12 and 13 (Med12 and Med13). However, Med12 and -13 do not associate with the MTBP-bound fraction of Cdk8/19-cyclin C: whereas anti-Cdk8 antibodies co-purified MTBP, Med12, and Med13 (Fig 5E), the same amount of Cdk8 purified with anti-MTBP antibodies did not contain Med12 and Med13 detectably. We conclude that Cdk8/19-cyclin C forms distinct protein complexes with the mediator of transcription and the origin firing regulator Treslin/TICRR-MTBP-TopBP1.

### Cdk8/19-cyclin C binding to MTBP requires the metazoa-specific MTBP middle domain

We then mapped the interaction site for Cdk8/19-cyclin C in MTBP-A516–K904. Binding studies using truncated MTBP fragments in cell lysates showed that amino acids R595 to P704 of MTBP are required and sufficient for binding the Cdk8 kinase (Figs 5F and S12A and S12B). The R595–P704 region contains a particularly well-conserved metazoa-specific domain (Fig 5H; blue oval; amino acids 636–693, S12D Fig). Deleting only this domain (MTBP-ΔT635–P704) abrogated Cdk8 binding, as did a mutant lacking amino acids R595 to L634 (Fig 5F). A series of MTBP point mutants subsequently showed that amino acids between L601 and E605 as well as L620 and T635 were important for MTBP binding to Cdk8/19-cyclin C (Fig 5G and S12C). In order to create a maximally Cdk8/19-cyclin C binding-deficient MTBP mutant for subsequent functional analyses, we combined seven amino acid exchanges between amino acids L620 and T635 to generate MTBP–Cdk8 binding mutant (Cdk8bm) (Fig 5G). MTBP-Cdk8bm bound Treslin/TICRR normally and behaved like MTBP-WT in gel filtrations (S3 Fig) and nuclear localisation (S4 Fig).

### Cdk8/19-cyclin C binding to MTBP is required for normal DNA replication in human cells

**Fig 6:**
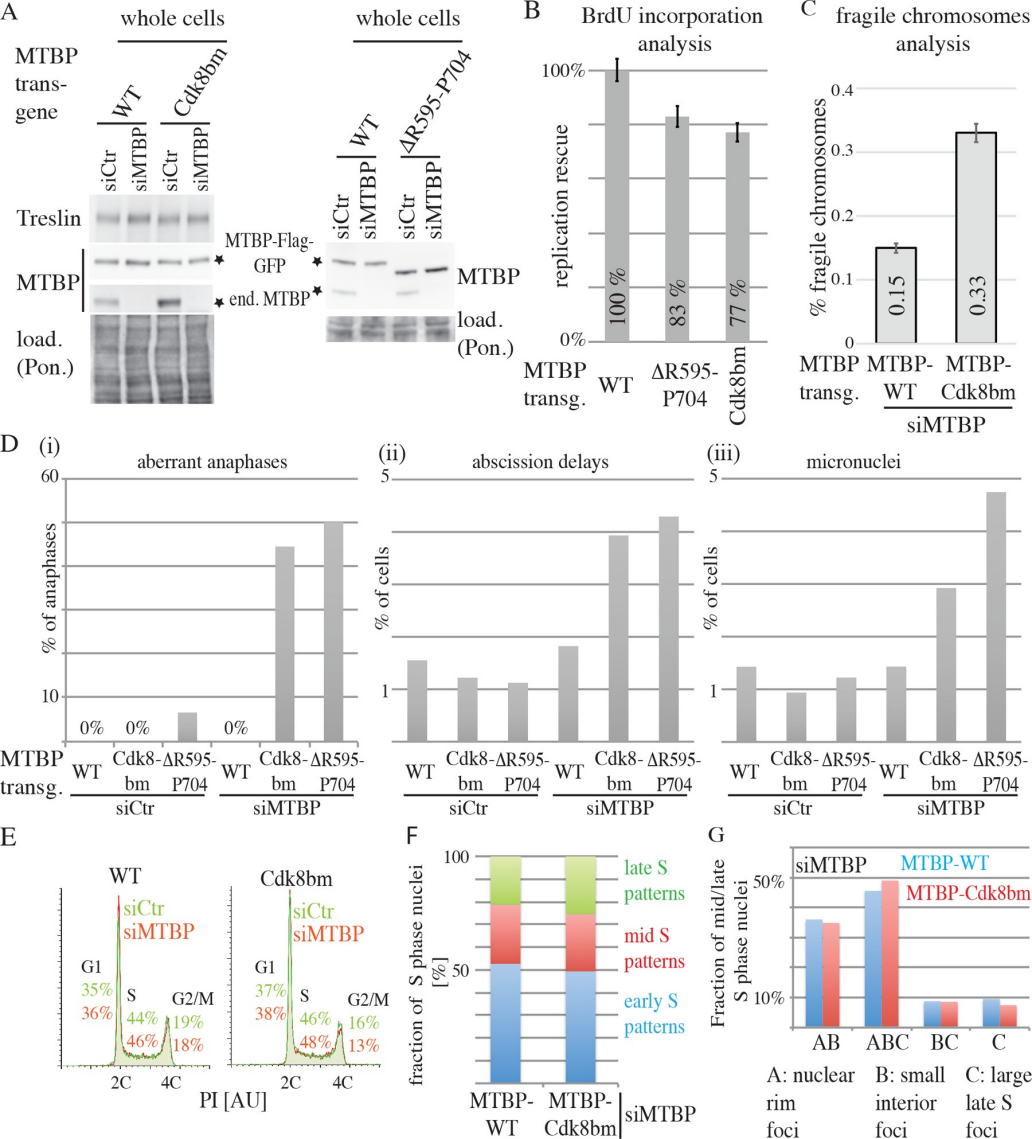
Cdk8/19-cyclin C binding to MTBP is required for proper genome replication in human cells. (A) Immunoblots of whole cell lysates of siCtr- or siMTBP-treated HeLa Flip-In T-Rex cells expressing MTBP-WT-Flag3-TEV2-GFP, MTBP–Cdk8bm-Flag3-TEV2-GFP, or MTBP-ΔR595–P704-Flag3-TEV2-GFP using anti-MTBP (4H9) and anti-Treslin (30E7). Ponceau (Pon.) staining controlled for loading. (B) Quantification of replication rescue of cells described in (A). Six (WT, Cdk8bm) or three (ΔR595–P704) independent experiments were analysed as in Fig 3G. Error bars: SEM. (C) Quantification of fragile chromosomes frequency in cells treated as in (A). Three independent experiments were analysed. Error bars: SEM. (D) Quantifications of aberrant anaphase figures, delays in cytokinetic abscission, and micronucleus frequency are shown. Cells were treated as in (A), fixed, and stained with anti-tubulin antibodies and dsDNA stain (Hoechst 33258) for fluorescence microscopy. (E) PI flow cytometry histogram plots of cells treated as described in (A). G1, S, and G2/M indicate fraction of cells in the respective cell cycle phase. (F) Early, mid–, and late–S phase replication patterns of cells described in (A) that stably expressed GFP-PCNA were quantified using live cell microscopy. (G) Experiment as in (F) was analysed for the indicated mid–and late–S phase–specific replication structures. AU, arbitrary units; BrdU, 5-bromodeoxyuridine; Cdk8/19, cyclin dependent kinase 8/19; Cdk8bm, Cdk8 binding mutant; dsDNA, double stranded DNA; GFP, green fluorescent protein; load., loading; MTBP, Mdm2 binding protein; PCNA, proliferating cell nuclear antigen; PI, propidium iodide; Pon., Ponceau; siCtr, control RNAi; siMTBP, MTBP-RNAi; WT, wild-type.

Next, we asked whether Cdk8/19-cyclin C, which is not amongst the yeast core firing factors, is a novel replication initiation factor in vertebrate cells. Because Cdk8 and Cdk19 may act redundantly, we knocked down cyclin C (siCycC; cyclin C siRNA) to test an involvement in DNA replication. BrdU–flow cytometry analysis revealed a mild reduction of replication speed in siCycC-treated HeLa-Kyoto and also in U2OS cells (S13A–S13C Fig and S1 Data). Because Cdk8/19-cyclin C regulates the transcription of many genes as part of the mediator complex, eliminating Cdk8/19-cyclin C from cells will generate complex phenotypes, leading to difficulties in differentiating primary and secondary defects. Therefore, we sought to separate mediator-dependent from -independent replication functions of Cdk8/19-cyclin C. To this end we tested if the binding of the Cdk8/19 kinase to MTBP is required for DNA replication. Because MTBP does not interact with mediator subunits (Fig 5E), Cdk8 kinase nonbinding MTBP mutants should reflect mediator-independent functions of the kinase. BrdU-incorporation analysis of siMTBP-treated cells expressing the Cdk8/19-cyclin C nonbinding MTBP-Cdk8bm and -ΔR595–P704 mutants showed a reduction of replication rescue of around 23% to 77% and 17% to 83%, respectively, compared with MTBP-WT (Fig 6A and 6B and S1 Data). This decrease of replication was more moderate than in siCycC cells (S13B and S13C Fig and S1 Data).

We sought to confirm this mild replication reduction by a more sensitive method. Compromised replication typically increases the frequency of fragile metaphase chromosome sites (FS). FS are under-replicated chromosome regions. A control experiment confirmed that FS frequency can be used to monitor replication defects by lower MTBP activity. HeLa cells treated with siMTBP, which reduces replication severely, displayed 5.6-fold more FS than siCtr-treated cells (S13D Fig and S1 Data). siMTBP-treated cells expressing the Cdk8/19-cyclin C binding-deficient MTBP mutants showed about two times more FS than siCtr-treated cells and siMTBP-treated cells expressing MTBP-WT (Fig 6C and S13E and S13F and S1 Data). We conclude that cells expressing Cdk8/19-cyclin C binding-deficient MTBP enter mitosis with incompletely replicated chromosomes. Such premature mitotic entry should increase aberrant mitotic figures. We fixed and stained siCtr- or siMTBP-treated MTBP-WT and Cdk8 binding-deficient cell lines with anti-tubulin antibodies and double stranded DNA (dsDNA) stain to visualise mitotic spindles and chromosomes. siMTBP-treated MTBP-Cdk8bm and -ΔR595–P704 cells showed more anaphases with DNA that was not pulled to the cell poles together with the remaining chromatin mass (Fig 6D(i) and S1 Data). Subsequent cytokinetic abscission was delayed, as judged by more cells that had already formed interphase nuclei but that were still connected by a thin connection containing the spindle midbody (ii). Delayed abscission upon incomplete replication has been described [36]. Occasionally, these connections contained Hoechst-positive structures. In line with chromosome segregation errors, we also found a higher frequency of micronuclei in MTBP-Cdk8bm and -ΔR595–P704 cells (iii). MTBP-ΔR595–P704 appears to have slight dominant effects, as suggested by the mutant cell lines showing a minor increase of aberrant anaphases upon siCtr treatment. We did not observe a compromised spindle assembly checkpoint response of MTBP-Cdk8bm and -ΔR595−P704 cells, as was reported for MTBP knock-down cells [37]. Nevertheless, we cannot exclude that a direct role of MTBP in mitosis contributes to the observed phenotypes.

That the observed DNA replication defect of cells expressing Cdk8/19-cyclin C nonbinding MTBP was mild was confirmed by the similar cell cycle distributions of siMTBP-treated MTBP-Cdk8bm and MTBP-WT cells (Fig 6E and S1 Data). Also, the temporal replication program was not grossly affected in MTBP-Cdk8bm cells. Classification of replication patterns using GFP–proliferating cell nuclear antigen (PCNA) in live cells expressing MTBP-WT and -Cdk8bm showed a normal appearance and order of early, mid–, and late–S phase replication patterns (Fig 6F and S1 Data). Also, in-detail inspection of mid–and late–S phase patterns revealed no differences (Fig 6G and S1 Data).

We conclude from Fig 6 that cells expressing Cdk8/19-cyclin C nonbinding MTBP mutants have moderate defects in genome replication and enter mitosis prematurely with under-replicated chromosomes.

The metazoa-specific MTBP middle region appears to harbour at least one more activity in addition to Cdk8/19-cyclin C binding that is required for replication. This activity is situated outside amino acids R595 to P704. MTBP-Cdk8bm and -ΔR595–P704, which are both maximally Cdk8/19-cyclin C binding deficient, show less severe defects in replication (around 20% reduction) than the mSld7 mutants (around 70%) that have larger deletions in the middle domain.

## Discussion

Our characterisation of MTBP as an origin firing factor that integrates conserved and metazoa-specific initiation processes presents an early step into understanding the specifics of higher eukaryotic replication in a field that has focused on showing that the principles of origin firing are conserved with yeast. We establish MTBP as the metazoan orthologue of yeast Sld7. Furthermore, we describe that MTBP also has metazoa-specific features that are important for replication. We identified Cdk8/19-cyclin C as a novel interactor of the metazoa-specific middle domain of MTBP and discovered that this binding is required for proper replication in human cells. Our work suggests that MTBP and its partners Treslin/TICRR-MTBP and TopBP1 function as a platform that integrates evolutionarily conserved and metazoa-specific replication-regulating signals to mediate complete and accurate replication of the genome.

### The presence of homologous and metazoa-specific regions implies that MTBP has conserved and specific replication functions

The orthology between MTBP and Sld7 described here is the final proof that all core factors for origin firing are conserved across fungi and animals (S14 Fig). The set of eukaryotic factors sufficient for conversion of pre-RCs into bidirectional replisomes with leading and lagging strand synthesis was defined by biochemical reconstitution with yeast proteins [16, 38, 39]. Whether these factors are also sufficient in higher eukaryotes now requires in vitro reconstitution of metazoan initiation from purified components.

Despite conservation of the core origin firing factors in eukaryotes, diversions of origin firing process occur in some species, indicating some evolutionary plasticity. For example, in fission yeast and *Caenorhabditis elegans*, Sld7/MTBP has not been found. Furthermore, the TopBP1 equivalent of the BRCT3/4 repeat domain of yeast Dpb11 that facilitates origin firing by interacting with CDK-phosphorylated Sld2 does not have an essential replication role in *Xenopus* egg extracts [24]. Instead, TopBP1-BRCT3 is essential [24]. Its role is unknown but it probably does not bind CDK-phosphorylated recombination helicase Q like 4 (RecQ4), metazoan Sld2, because TopBP1-BRCT3 lacks key amino acids for phosphopeptide binding [40].

Metazoan cells are more complex than yeasts and will probably require more sophisticated regulation of replication initiation. The metazoa-specific domains of MTBP and of other firing factors like Treslin/TICRR [25] may help integrate the conserved fundamental initiation processes into metazoan cells. Metazoa have less precisely defined origins than yeast, but they face the same problems as any cell with multiple replication start sites. Genome duplication needs to be complete before mitosis to minimise genome instability. This necessitates that no inter-origin distance is bigger than the amount of DNA that can be replicated in S phase. To avoid large random gaps, appropriately located origins must fire with the right timing. The organisation of metazoan replication in replication factories, in which neighbouring origins fire with near synchrony, may help prevent large random gaps [41]. The factory organisation may also help facilitate differential regulation of origin firing between different factories upon DNA damage. In these circumstances so-called dormant origins (that are inactive in normal growth conditions) fire in actively replicating factories after DNA damage, whereas origin firing becomes inhibited in nonreplicating factories. Both regulations help prevent genetic instability upon replication stress [42].

MTBP is a promising candidate for mediating some of these regulations, as it forms a main regulation platform of origin firing in eukaryotes together with Treslin/TICRR/Sld3 and TopBP1/Dpb11. All complex members are subject to regulations by various pathways to mediate appropriately timed and localised replication initiation. These regulations mediate (1) S phase specificity of replication via CDK and DDK [5, 8, 9], (2) replication timing of centromeres and other genome regions [43–45], (3) inhibition of origin firing upon DNA damage by S phase checkpoint kinases [46–48], and (4) firing inhibition in response to low cellular glucose levels involving the silent information regulator (SIRT1) deacetylase [49, 50]. These regulations all involve Sld3/Treslin/TICRR, Dpb11/TopBP1, or Sld2/RecQ4, and at least some are conserved between yeast and man [26, 27, 51]. How MTBP activity is controlled is unknown. Several posttranslational modification sites in MTBP were identified by high-throughput approaches but have not been functionally investigated, among them potential ataxia teleangiectasia mutated related/mutated (ATR/M) and CDK sites. These are good candidates to mediate cell cycle and checkpoint regulations. Cdk8/19-cyclin C binding to MTBP could also be involved in mediating firing control (see below).

### Roles of the Sld7-homologous MTBP domains

We show that the S7M-N domain of MTBP mediates Treslin/TICRR binding. As with yeast Sld7 and Sld3, complex formation of MTBP with Treslin/TICRR is important for MTBP’s replication function, as several nonoverlapping point and deletion mutants of MTBP that are deficient in Treslin/TICRR binding failed to support replication in HeLa cells. The same was true for MTBP interaction-deficient mutants of Treslin/TICRR [28]. Treslin/TICRR binding-deficient MTBP mutants, for example MTBP-5m and m1–6, showed reduced MTBP levels in cell lysates and had lower levels of endogenous Treslin/TICRR. The lower levels of the MTBP mutants were at least partly due to their higher degradation rate compared with MTBP-WT (S8A and S8B Fig). This corroborates earlier RNAi experiments suggesting that Treslin/TICRR and MTBP stabilise each other’s cellular levels [28] (S8C and S8F Fig and S1 Data): Treslin/TICRR RNAi reduced MTBP levels and vice versa, and this was rescued by RNAi-resistant proteins. Mutual stabilisation is also seen with Sld3 and Sld7, and it is important for replication in yeast [17]. Lower Treslin/TICRR levels may make a small contribution to the replication defects in human cells expressing Treslin/TICRR nonbinding MTBP mutants. However, Treslin/TICRR stabilisation cannot be the only function of MTBP in replication, because (1) deleting the MTBP middle and the S7M-C domains results in a fully Treslin/TICRR–binding-proficient MTBP1-515 protein that is, however, incapable of inducing replication over background, (2) elevating the expression levels of the non-Treslin/TICRR–binding MTBP mutants to MTBP-WT did not rescue replication in human cells, (3) restoring Treslin/TICRR levels in the absence of MTBP binding was insufficient to restore replication in Treslin siRNA (siTreslin)–treated cells [28] and in MTBP-immunodepleted *Xenopus* egg extracts [29], and (4) our partial RNAi-depletion experiments with Treslin/TICRR showed that strong reduction of replication—as observed in MTBP-5m and m1–6 cell lines—is only achieved by depleting Treslin/TICRR to levels well below the levels in these MTBP mutant lines (S8A, S8E and S8F Fig and S1 Data).

The S7M-C domain of MTBP—albeit not essential for replication—is required for normal replication levels in HeLa cells. Its function is distinct from Treslin/TICRR binding because MTBP-ΔS7M-C bound Treslin/TICRR as well as MTBP-WT. In yeast, the S7M-C domain mediated Sld7 dimerisation in the crystal structure to form dimers of Sld3-Sld7 heterodimers [18]. How important Sld7 dimerisation is for replication in yeasts was not addressed. We found that attaching an artificial dimerisation domain in the form of GST to S7M-C deletion mutants of MTBP rescues the ability of these mutants to induce replication in Hela cells. Sld3/Treslin-Sld7/MTBP dimerisation could help ensure that both MCM helicases in pre-RCs become activated, but never only one, which could generate initiation intermediates that jeopardise genetic stability. We cannot formally exclude that GST rescues replication in cells expressing S7M-C MTBP deletion mutants by virtues other than homodimerisation that it does not share with the GFP control tag. We found no biochemical evidence of S7M-C–dependent MTBP dimerisation by binding studies using pulldowns with MTBP fragments expressed in 293T cells. This indicates that S7M-C–mediated MTBP dimerisation may be weak or spatiotemporally regulated, e.g., by posttranslational modification.

### Role of CDK8/19-cyclin C binding to MTBP in replication

Three lines of evidence support the conclusion that Cdk8/19-cyclin C has a role in genome replication that requires its binding to the metazoa-specific middle domain of MTBP. (1) Cdk8/19-cyclin C forms a protein complex with the MTBP-Treslin/TICRR-TopBP1 replication initiation regulator. (2) Cells expressing deletion or point mutants of MTBP that are deficient in binding to Cdk8/19-cyclin C show compromised replication. (3) RNAi depletion of cyclin C also compromises replication.

We believe that the function of Cdk8/19-cyclin C in replication is distinct from its classic role as the kinase module of the mediator of transcription because Cdk8/19-cyclin C forms distinct complexes with MTBP-Treslin/TICRR-TopBP1, or the kinase module of the mediator subunits Med12 and Med13. Moreover, in contrast to RNAi depletion of cyclin C knock-down of Med12, which shares many mediator-related functions with Cdk8/19-cyclin C [52, 53], did not lead to replication defects (S13G and S13H Fig and S1 Data).

The exact role in the replication of Cdk8/19-cyclin C in complex with MTBP-Treslin/TICRR-TopBP1 is unclear. Cdk8/19-cyclin C nonbinding MTBP mutants replicate slightly slower than MTBP-WT cells. Apparently, the replication problems in the mutant cell lines are severe enough to cause unreplicated gaps in the genome that become evident as fragile metaphase chromosomes and aberrant mitotic figures. Compromised replication was previously shown to induce a checkpoint that delays cytokinetic abscission [36]. This checkpoint is thought to provide cells in which abscission is not possible normally, with an opportunity to nevertheless achieve division and avoid tetraploidy [54, 55]. Consistently, we find delayed abscission in our mutant MTBP cell lines.

Due to these phenotypic analyses, we believe that insufficient replication forks are generated in cells with Cdk8/19-cyclin C binding-deficient MTBP, resulting in replication gaps. One model is that Cdk8/19-cyclin C binding to MTBP is generally required for maximal activity of MTBP to promote origin firing. Alternatively, a specific subset of firing events could be controlled by Cdk8/19-cyclin C. This could be a positive control, so that inefficient firing of CDK8-activated origins causes replication gaps, or a negative control, so that ectopic firing of CDK8-inhibited origins causes replication gaps indirectly, for example, by promoting replication-transcription collisions or by depleting certain genomic regions of dormant origins. We cannot exclude that Cdk8/19-cyclin C binding to MTBP has roles in transcription or other processes that contribute to the observed phenotype.

## Materials and methods

### Cell culture

All cell lines were maintained in DMEM (Life Technologies) supplemented with 10% FBS (Life Technologies) and 1% penicillin/streptomycin antibiotics (Life Technologies) in 5% CO_2_ conditions at 37°C.

Stable MTBP transgene–expressing HeLa Flip-In T-Rex cell lines [56] were generated according to the manufacturer’s instructions (Invitrogen) using pcDNA5-FRT-TO-Flag3-Tev2-GFP or pcDNA5-FRT-TO-Flag3-Tev2. Cell pools containing at least 50 individual clones were used to average out clonal variation.

Stable MTBP transgene-expressing HeLa Kyoto cell lines were generated using pIRESpuro3-Flag-AcGFP.

For cell synchronisations, HeLa-Flp-In T-Rex or U2OS cells were treated with 2 mM thymidine for 24 h. For S phase populations, double thymidine-arrested cells were harvested in thymidine or released for 3 h before harvesting. G2/M phase populations were released from thymidine arrest for 10 h and for G1 phase populations for 14 h. For release, cells were washed twice in PBS before incubation in normal medium. For mitotic blocks, unsynchronised cells or cells released from thymidine were treated with 0.1 μg/mL nocodazole overnight before harvesting by mitotic shake-off. For nocodazole release into G1 phase, mitotically arrested cells were washed twice in PBS before incubation in medium lacking nocodazole. Only attached cells were harvested by trypsinisation for G1-synchronised populations.

### Transient transfection of 293T cells

Cells (293T) were transfected using standard calcium phosphate transfection. In brief, an approximately 60%–70% confluent 10-cm dish of 293T cells was transfected with 13 μg plasmid DNA using 83 μL 2 M CaCl_2_, 566 μL sterile H_2_O, and 667 μL 2×HBS (50 mM HEPES, 10 mM KCl, 2 mM dextrose, 280 mM NaCl, 1.5 mM Na_2_HPO_4_). The medium was changed 24 h after transfection, and cells were cultivated for another 48 h before harvesting.

### RNAi

HeLa Flip-In T-Rex cell lines carrying transgenes or control cells were treated with 1 μg/mL doxycycline roughly 2 h prior to transfection to induce expression. siRNA transfections were carried out using RNAiMAX (Life Technologies) according to the manufacturer’s guidelines. In brief, 1.5 × 10^5^ cell per 6-cm tissue culture dish were transfected with 20 nM control siRNA (GL2) or siRNA against MTBP (1:1 mix of MTBP1 and MTBP2) using 10 μL RNAiMAX in 5 mL volume.

For MTBP and Treslin/TICRR immunoblots following RNAi, whole cells were boiled in 3× Laemmli sample buffer, separated by SDS-PAGE, and blotted on nitrocellulose membranes before detection with anti-MTBP and anti-Treslin/TICRR antibodies.

### Cell cycle flow cytometry

Seventy-two hours after RNAi transfection, cells were pulse labelled using 10 μM BrdU for 30min. Cells were then harvested and fixed with −20°C cold methanol for 24 h. The fixed cells were treated with 2 M HCl/0.5% Triton X-100 for 30 min before washing extensively with PBS/0.5% Triton. Incorporated BrdU was detected using FITC-coupled mouse anti-BrdU in 1% BSA/PBS/0.01% Triton X-100 for 1 h according to the manufacturer’s instructions. After washing once with PBS/0.01% Triton X-100, the DNA was stained with 25 μg/mL PI in the presence of 100 μg/mL RNaseA. Flow cytometry analysis was performed using a FACSCalibur flow cytometer (BD Biosciences) with a logarithmic setting of the FL1-H channel for FITC detection and a linear setting of the FL2-H channel for PI detection. Data analysis was performed using the Kaluza Analysis 1.3 software (Beckman Coulter). Cell aggregates were excluded based on their high FL2-W signal. BrdU-PI profiles were generated as density plots. Reduction of BrdU incorporation upon siRNA treatment was analysed by gating S phase cells in BrdU/PI dot plots and visualised as BrdU histogram overlays. For quantifications of the efficiency of replication rescue by MTBP transgenes, the BrdU signal of BrdU-positive S phase cells was background-subtracted using the signal intensity of BrdU-negative G1 and G2/M phase cells to calculate the replication-dependent BrdU signal. This value was divided by the BrdU replication signal of control siRNA–treated cells. Visualisation as bar diagrams and statistics was done using GraphPad Prism 5 (GraphPad Software).

### Fragile chromosome site analysis

To assess the frequency of fragile chromosomes, cells were treated for 2 h with 0.08 μg/mL colcemide. After mitotic shake-off, cells were treated with 1% sodium citrate for 10 min and fixed by washing three times with methanol/acetic acid (3:1). Droplets of cells in fixation solution were dropped on glass slides from a height of about 150 cm. After drying at RT for 24 h, cells were Giemsa stained using 1:20 Giemsa (Merck 109204) in 4 mM NaHPO_4_, 5 mM KHPO_4_. Fragile chromosomes were imaged using conventional light microscopy (40× oil lens) using the Zeiss ZEN 2.3 software. The fractions of fragile chromosomes were assessed manually. For quantification, around 8,000 chromosomes per sample were analysed.

### Live cell microscopy

HeLa Flip-In T-Rex cell lines stably expressing AcGFP-PCNA were seeded 24 h after siRNA treatment into 4-well glass bottom plates (ibidi 80427). Imaging was done using an Andor/Nikon spinning disk confocal microscope at 37°C and 5% CO_2_ in imaging medium (DMEM, Life Technologies) supplemented with 10% FBS, 1% penicillin/streptomycin antibiotics (Life Technologies). Excitation was at 475 nm laser wavelength. A frame rate of one image per 30 min was used for each 48-h imaging session. Three Z sections per frame were taken. For image processing, maximal intensity projections were made, and Fiji software was used to generate movies. Movies were analysed visually to assess replication patterns. To analyse the temporal replication program, S phase patterns were classified into early, mid–, and late–S phase patterns and quantified. Early S phases were from the first appearance of replication foci to the first frame, with mid–S phase patterns defined as nuclear rim and/or perinucleolar replication. Late patterns were all frames with distinctive large bright replication foci. Quantifications represent the fraction of early, mid, and late patterns of the total number of S phase frames. For detailed analysis of mid/late replication patterns, three classes of patterns were defined: (A) fragmented or complete nuclear rim staining, (B) small intranuclear foci, and (C) bright large foci in the nuclear interior or at the rim. Frames showing a single pattern or combinations of patterns were quantified and normalised to the total frame number.

### Mitosis analysis using fixed cell fluorescence microscopy

Cells on glass coverslips were fixed with 4% paraformaldehyde for 10 min at RT. After permeabilisation and blocking in PBS, 0.1% BSA, 0.1% Triton X-100 for 1 h, anti-tubulin and anti-mouse Alexa 488 antibodies were used at dilutions of 1:2,000 and 1:100, respectively, in blocking buffer (PBS, 0.1% BSA) in distinct 1-h incubation steps to stain tubulin. A total of 1 μg/mL dsDNA stain (Hoechst 33258; Sigma, B2883) in blocking buffer was used to stain DNA. After mounting in FluorSave Reagent (Calbiochem, 345789), cells were imaged using a Zeiss Axio Observer7 microscope. Eleven Z sections were taken and analysed visually using the Fiji software. Anaphases were classified as ‘aberrant’ if they showed a DNA signal between the separating DNA masses. For quantification, aberrant anaphases were normalised to the total number of anaphases. Cells with cytokinesis delays showed a thin connection containing a spindle midbody between two daughter cells that had already formed clear interphase nuclei with decondensed chromatin. Micronuclei were defined as DNA masses in interphase cells that were clearly distinct from the main nucleus. For quantification of cytokinesis delays and micronuclei, their number was normalised to the total number of cells.

### Immunofluorescence to determine subcellular MTBP localisation

Hela Flip-In cells were grown on glass coverslips. Transient transfection was performed using Lipofectamine 2000 (Life Technologies) according to the manufacturer’s instructions. Cells were fixed 48 h after transfection using 4% paraformaldehyde for 15 min at RT. After three washing steps with PBS, cells were permeabilised and blocked with PBS, 0.1% BSA, 0.1% Triton X-100 for 1 h at RT. Subsequently, cells were incubated for 1 h at RT with the primary antibodies (anti-FLAG M2 1:1,000 or anti-GFP 1:250) diluted in blocking buffer. After three washing steps using PBS with 0.1% BSA, cells were incubated with anti-mouse Alexa 488 (1:250) and 1 μg/mL dsDNA stain (Hoechst 33258; Sigma, B2883) for 1 h at RT. After three washing steps and mounting using Roti-Mount FluorCare (Carl Roth, HP19.1), image acquisition was performed using the Zeiss Axio Observer 7.

#### IP

For coimmunoprecipitations of tagged Treslin/TICRR and MTBP from 293T cells, a 10-cm plate of transiently transfected 293T cells were lysed in 5× pellet volume of lysis buffer (20 mM HEPES, 150 mM NaCl, 10% glycerol, complete EDTA-free protease inhibitor cocktail [Roche, 05056489001], 0.1% Triton X-100, 2 mM 2-mercaptoethanol). For flag affinity purifications 50% of cell lysates was incubated with 1 μg anti-FLAG mouse monoclonal antibody or 1 μg mouse IgG coupled to 150 μg Protein G Dynabeads (Invitrogen, 100-04D). For anti-GFP pulldowns, lysates from one 10-cm plate of transfected cells were prepared in lysis buffer with 250 mM NaCl and incubated with 20 μg anti-GFP nanobodies covalently coupled to 10 μL NHS sepharose (GE Healthcare, 10343240). After incubation for 1 h at 4°C, the immunoprecipitates were washed with lysis buffer and retained proteins eluted by boiling in Laemmli sample buffer. The samples were analysed by immunoblotting.

IPs of endogenous MTBP, TopBP1, or Cdk8/19-cyclin C, the respective antibodies, or control IgGs were coupled to Protein G Dynabeads and incubated in native cell lysates (lysis buffer 20 mM HEPES, 150 mM NaCl, 10% glycerol, complete EDTA-free protease inhibitor cocktail, 0.1% Triton X-100, 2 mM 2-mercaptoethanol, 5 μg/mL cytochalasin D [Sigma, C8273]) for 2 h at 4°C. After washing, bead-bound proteins were eluted by boiling in Laemmli buffer. For IP from CDK-treated lysates, native lysates were incubated with CyclinA-Cdk2 (a generous gift of Tim Hunt) and 5 mM ATP/5 mM MgCl_2_ for 5 min at 25°C before the addition of beads.

### Antibodies

For generation of the monoclonal antibody against human Treslin/TICRR (clone 30E7: N-His-Treslin [amino acids 260–791]), MTBP (clone 12H7: N-His-MTBP [amino acids M1–I284]), and clone 4H9 (N-His-MTBP [amino acids F102-Y513]), approximately 50 μg of His-tagged fusion protein dissolved in PBS was emulsified in an equal volume of incomplete Freund’s adjuvant, and Wistar rats (MTBP antigens) or C57BL/6J mice (Treslin antigen) were immunised subcutaneously (s.c.) and intraperitoneally (i.p.). Six weeks after immunisation, a 50-μg boost injection was applied i.p. and s.c. 3 d before fusion. Fusion of the splenic B cells and the myeloma cell line P3X63Ag8.653 was performed using polyethylene glycol 1500 according to standard protocols [57]. Hybridoma supernatants were tested by solid-phase ELISA using the His-fusion proteins and verified by western blotting. Monoclonal hybridoma cell lines of the Treslin/TICRR- and MTBP-reactive supernatants were cloned twice by limiting dilution. The IgG subclasses were determined with ELISA assay as MTBP clones 12H7 and 4H9: rat IgG2b and Treslin/TICRR clone 30E7.

### Treslin/TICRR domain mutants

The following nomenclature was used for Treslin/TICRR domain mutants: ΔCIT, the N-terminal 264 amino acids of Treslin/TICRR were deleted; ΔM1, amino acids 265–408 were deleted; ΔM2, amino acids 409–593 were deleted; ΔSTD, amino acids 717–792 were deleted; 2PM, the two essential CDK sites (T969A; S1001A) were mutated to alanine; ΔC-terminal, The C-terminus was deleted from amino acid 1057.

### Gel filtration

Overexpressed Flag-purified MTBP-3Flag was loaded onto a Superdex 200 column (2.4 mL 3.2/300). FPLC was performed at +4°C at a 30 μL/min flow rate with a 100-μL fraction size in running buffer (20 mM HEPES, pH 8.0, 200 mM NaCl, 0.5 mM TCEP, 10 mM NaF, 0.1% TritonX-100). A total of 1/15 of each fraction was analyzed via western blot. Gel filtration standard (1511901, BioRad) was used to determine apparent molecular weights.

### Chromatin preparation

Chromatin-enriched fractions were purified from Hela Flp-In cells via lysis in 10 mM HEPES, pH 7.0, 100 mM NaCl, 300 mM sucrose, 3 mM MgCl_2_, 0.5% Triton, 5mM ß-mercaptoethanol, complete EDTA-free protease inhibitor cocktail, and 5 μg/mL cytochalasin D. Chromatin was harvested by centrifugation at 3,200 rpm for 4 min at 4°C in table-top centrifuge and washing the chromatin pellet in lysis buffer three times, involving 5 min of incubation in washing buffer.

## Supporting information

S1 Fig A Sld7-homologous region in the N terminus of MTBP identified using phyre2 (MTBP-phyr2 region). (A) The phyre2 server was queried with MTBP sequences from a selection of metazoan MTBP sequences (human, cow, fish, mouse, frog, octopus). Phyre2 returned alignments between MTBP from cow and fish (*Cyprinodon variegatus*) with yeast Sld7 with low confidence scores of 11.4% and 13.0%. Numbers are amino acid positions. (B) T-coffee alignment between the indicated MTBPs from selected vertebrates and molluscs, illustrated using Jalview. Colouring of amino acids indicates similarity according to blocks substitution matrix 62 (BLOSUM62) score. Amino acid positions 305, 310, and 320 in hMTBP; blue asterisks, amino acids contacting Sld3 as indicated in (A) [18]; 0–9, +, * indicate relative conservation, with 0 not conserved and * fully conserved. BLOSUM62, blocks substitution matrix 62; hMTBP, human MTBP; MTBP, Mdm2 binding protein; phyre2, protein homology/analogy recognition engine; pred, predicted; Sec. str., secondary structure; Sld7, synthetic lethal with Dpb11 7.
(TIF)

S2 Fig Sequence homology of the N termini of MTBP and Sld7. (A) Full sequence alignment, of which parts are shown in Fig 1C, of the S7M-N domains of MTBP and Sld7. In Fig 1C, only the two regions in the S7M of Sld7 that interact with Sld3 are shown. Direct comparison of the N-terminal domain profiles of metazoan MTBP and fungal Sld7 yielded an *E*-value of 3.0 × 10^−5^ (probability: 78.5%). The phyre2 region and the region containing the second cluster of Sld3-interacting amino acids (C413–G426) are indicated. Asterisks mark the positions of Sld3-interacting amino acids in yeast Sld7 and the corresponding positions in MTBP. (B) Critical Treslin/TICRR-binding amino acids in MTBP map to Sld7 residues that directly contact Sld3 in the crystal structure of the Sld7-Sld3 dimer [18]. MTBP, Mdm2 binding protein; Sld7, synthetic lethal with Dpb11 7; S7M-N, Sld7-MTBP N-terminal domain; TICRR, TopBP1 interacting checkpoint and replication regulator.
(TIF)

S3 Fig Mutant and WT MTBP proteins fractionate similarly in gel filtrations. Flag-tagged MTBP-WT and indicated mutant MTBP proteins were isolated by Flag IP and Flag peptide elution from lysates of transiently transfected, and therefore highly overexpressing, 293T cells. Separation by gel filtration using a Superdex 200 column (3.2/300; 2.4 mL) followed. All MTBP versions eluted in distinct peaks, and similar fractions of the input were recovered, indicating stable folding. MTBP-Δ595–704 eluted slightly later from the column due to its smaller size. MTBP, Mdm2 binding protein; WT, wild-type.
(TIF)

S4 Fig Subcellular localisation of mutant MTBP proteins. Hela Flp-In cells were transiently transfected with the indicated Flag- or GFP-tagged MTBP-WT and mutants, and then immune-stained with anti-Flag or anti-GFP, as indicated. ‘none’ indicates nontransfected cells. dsDNA staining (Hoechst 33258) served to mark the nuclear DNA. dsDNA, double stranded DNA; GFP, green fluorescent protein; MTBP, Mdm2 binding protein; WT, wild-type.
(TIF)

S5 Fig Treslin/TICRR in cell lysates and the MTBP-bound fraction of Treslin/TICRR are phosphorylated. Cell lysates of Hela cells (in 20 mM HEPES, 150 mM NaCl, 10% glycerol, complete EDTA-free protease inhibitor cocktail, 0.1% Triton X-100, 2 mM 2-mercaptoethanol expressing MTBP-WT-3Flag) were treated with lambda PPase (8,000 units in 1mL lysate; NEB P0753) or buffer according to the manufacturer’s instructions. Lysates were then used for Flag IPs with 40 uL slurry of M2 anti-Flag magnetic beads (M8823, Sigma) to isolate MTBP-Flag and associated Treslin/TICRR. Lysates and IPs were separated on a 3%–8% Tris-acetate Criterion gel (BioRad) for optimal resolution. Immunoblotting for Treslin/TICRR and MTBP showed that the gel mobility of Treslin/TICRR shifts in PPase treatment in lysates, indicating phosphorylation. In the absence of PPase, Treslin/TICRR in MTBP-bound Treslin/TICRR showed indistinguishable running behaviour from Treslin/TICRR in lysates, indicating phosphorylation of MTBP-bound Treslin/TICRR. IP, immunoprecipitation; MTBP, Mdm2 binding protein; PPase, phosphatase; TICRR, TopBP1 interacting checkpoint and replication regulator; WT, wild-type.
(TIF)

S6 Fig The replication-inducing activity of several single MTBP point mutants in the MTBP-S7M-N domain correlates with their Treslin/TICRR-binding capability. (A) Immunoblot showing MTBP levels in lysates of siCtr of siMTBP-treated cells expressing siRNA-resistant MTBP-WT-3Flag or mutants carrying the indicated single amino acid exchanges. The combination mutants 5m and m1–6, from which the single mutants were derived, are shown for comparison. In the long exposure, the stronger signals in siCtr lysates were in the saturated range. (B) Replication was quantified using BrdU incorporation and flow cytometry after replacing endogenous MTBP with RNAi-resistant MTBP-WT or mutants in stable Hela Flp-In cell lines. I309D, D313A, and L314D are single point mutations that were also mutated in the MTBP-5m combination mutant. I309D and L314D were defective in Treslin/TICRR binding, whereas D313A bound Treslin/TICRR like MTBP-WT (Fig 2A). A415Q, also mutated in the m1–6 combination mutant, was Treslin/TICRR-binding deficient, as was MTBP-ΔD2-V8 (Fig 2B). To show that the cells expressing Treslin/TICRR nonbinding mutants showed similarly low replication activity as siMTBP-treated cells without a transgene, the data were not normalised to no-transgene cells for this graph. Error bars: SEM from three independent experiments. BrdU, 5-bromodeoxyuridine; m, point mutation; MTBP, Mdm2 binding protein; RNAi, RNA interference; siCtr, control RNAi; siMTBP, MTBP-RNAi; siRNA, small interfering RNA; S7M-N, Sld7-MTBP N-terminal domain; TICRR, TopBP1 interacting checkpoint and replication regulator; WT, wild-type.
(TIF)

S7 Fig Control experiments for chromatin fractionation experiments (Fig 3D). (A) Origin licensing and firing can be assessed by immunoblotting of chromatin isolated from cells. For (i), chromatin was isolated from cells synchronised in mitosis, in G1 or S phase, using thymidine arrest and release for 3 h (S phase cells), 10 h (G2/M), or 14 h (G1). Mcm5 signals present in G1 chromatin, but less in mitosis, showed that licensing can be monitored. Cdc45 and Sld5 (GINS) were detected specifically on S phase chromatin, confirming these are adequate markers for origin firing. (ii) Shows PI-based cell cycle analysis by flow cytometry of samples used in (i). (B) Flow cytometry of the PI-stained cells described in Fig 3D showed that the cell cycle distribution was largely unchanged under the experimental conditions. Cdc45, cell division cycle 45; GINS, go-ichi-ni-san; Mcm5, minichromosome maintenance 5; PI, propidium iodide; Sld5, synthetic lethal with Dpb11 5.
(TIF)

S8 Fig Strong decreases of MTBP and Treslin/TICRR levels are required to elicit significant replication defects. (A) Quantification of immunoblot signals of samples shown in Fig 3A shows that Treslin/TICRR binding deficient MTBP mutants have only moderately decreased levels of MTBP and Treslin/TICRR. (B) Treslin binding-deficient MTBP is less stable than MTBP-WT. HeLa Flp-In T-Rex cells expressing MTBP-3×Flag-WT or the indicated Treslin/TICRR binding-deficient mutants were doxycycline induced overnight before shutoff of gene expression by doxycycline withdrawal and addition of 100 μg/mL cycloheximide for the indicated times. Whole cell lysates were then immunoblotted with anti-MTBP antibodies (4H9). The Treslin/TICRR binding deficient mutants decreased in levels within 1 h. In contrast, endogenous MTBP and MTBP-WT-3×Flag were largely stable for 4 h. (C,D) Strong down-regulation of MTBP is required to suppress replication. HeLa Flp-In T-Rex control cells were treated with increasing concentrations of siMTBP. Treslin/TICRR levels decrease moderately in MTBP-depleted cells. Whole cell lysates were immunoblotted to assess relative levels of MTBP and Treslin/TICRR (C). (D) Shows quantification of the immunoblot signals shown in (C) and of replication levels, as assessed by BrdU incorporation and flow cytometry. (D) Indicates that MTBP signals need to decrease by roughly 90% to achieve significant reduction of replication in these cells. (E,F) Strong down-regulation of Treslin/TICRR is required to suppress replication. MTBP levels strongly decline upon Treslin/TICRR depletion. The experiment described in (C, D) was repeated with siRNA against Treslin/TICRR. BrdU, 5-bromodeoxyuridine; MTBP, Mdm2 binding protein; siMTBP, MTBP-RNAi; siRNA, small interfering RNA; TICRR, TopBP1 interacting checkpoint and replication regulator; WT, wild-type.
(TIF)

S9 Fig Expression of Treslin/TICRR binding-deficient MTBP mutants at levels comparable to or higher than MTBP-WT does not result in high levels of DNA replication. Hela Kyoto cell lines with stably integrated siMTBP-resistant MTBP-WT-Flag-GFP, MTBP-5m-Flag-GFP (clones 1 and 2), or MTBP-m1–6-Flag-GFP in pIRES-puro3 were RNAi treated with siCtr or siMTBP. (A) Immunoblot showing the relative levels of the transgenes. (B) Replication was measured by BrdU incorporation and flow cytometry. S phase subpopulations of siCtr- and siMTBP-treated samples were overlaid and the fold difference of BrdU signal calculated (1.2–2.5×; note that numbers are not directly comparable with Fig 3C, F because of the different Hela cell lines used). In contrast to MTBP-WT, the -5m and -m1–6 mutants support DNA replication poorly despite equal (5m clone 1, m1–6) or higher (5m clone 2) expression levels. BrdU, 5-bromodeoxyuridine; GFP, green fluorescent protein; m, point mutation; MTBP, Mdm2 binding protein; pIRES, plasmid named internal ribosomal entry site; puro3, puromycin 3; RNAi, RNA interference; siCtr, control RNAi; siMTBP, MTBP-RNAi; TICRR, TopBP1 interacting checkpoint and replication regulator; WT, wild-type.
(TIF)

S10 Fig Cdk8 binding capacity and expression levels of MTBP-ΔC mutant proteins. (A) Mutants in the S7M-C domain of MTBP are proficient in Treslin binding. Native lysates of HeLa Flp-In T-Rex cells expressing MTBP-WT or mutants lacking the last 81 (ΔS7M-C) or 150 (ΔC150) amino acids were used for IP of endogenous Treslin using control IgGs (IgG-IP) or rabbit anti-Treslin-970-1400 antibodies (Tres-IP). Lysates and bead-bound material were then analysed in immunoblots using anti-MTBP and anti-Treslin antibodies. B) siMTBP-treated Hela Flp-In cell lines MTBP-WT-3Flag, MTBPΔC150, MTBPΔC150-GST, and MTBPΔC150-GFP were lysed in SDS sample buffer and tested by immunoblotting with anti-hMTBP (83) for expression levels of the transgenes. Cdk8, cyclin dependent kinase 8; GFP, green fluorescent protein; GST, glutathione S transferase; hMTBP, human MTBP; IgG, immunoglobulin; IP, immunoprecipitation; MTBP, Mdm2 binding protein; siMTBP, MTBP-RNAi; S7M-C, Sld7-MTBP C-terminal domain; WT, wild-type.
(TIF)

S11 Fig Treslin/TICRR binding capacity of various fragments and mutants of MTBP. (A) Treslin/TICRR binding activities of N-terminal MTBP fragments. hMTBP, full length, or the indicated fragments were translated in SP6 reticulocyte lysates (Promega L2080) in the presence of ^35^S-methionine, and HA-Treslin/TICRR-1-671 was translated in lysates without radioactive methionine. MTBP and Treslin/TICRR lysates were mixed, and IPs with unspecific IgG or anti-HA antibodies were made. Lysates (Inputs) and bead-bound material (IP) were analysed by autoradiography and anti-HA immunoblotting. (B) Binding of mSld7s to Treslin/TICRR. Lysates of Fig 4A were used for IPs with GFP nanobodies or mock-coupled control beads, and then analysed for bead-bound MTBP and Treslin/TICRR by immunoblotting with anti-MTBP (4H9) and anti-Treslin (148). GFP, green fluorescent protein; HA, hemagglutinin; hMTBP, human MTBP; IgG, immunoglobulin; IP, immunoprecipitation; mSld7, metazoan Sld7; MTBP, Mdm2 binding protein; SP6, SP6 virus; TICRR, TopBP1 interacting checkpoint and replication regulator.
(TIF)

S12 Fig Cdk8/19-cyclin C binds MTBP dependently on the region containing amino acids R595–P704 in the metazoan-specific MTBP part. (A) Schematic summarising MTBP fragment-based Cdk8/19-cyclin C binding studies shown in (B). (B) MTBP-WT or N- or C-terminal fragments of MTBP containing the indicated amino acids (aa) were transiently transfected into 293T cells. Subsequent IP from native lysates with the anti-GFP (i) or anti-Cdk8 (ii, iii) antibodies were analysed by immunoblotting using anti-MTBP (4H9), anti-Cdk8, anti-GFP, or anti-cyclin C antibodies. Overexp. indicates long exposures in which the stronger signals were saturated. (C) MTBP-Flag-GFP-WT or the indicated point mutants in the Cdk8/19-cyclin C binding region mapped in (A,B) were tested for interaction with endogenous Cdk8/19-cyclin C using transient transfection of MTBP into 293T cells and anti-GFP IP. Subsequent immunoblots were analysed with anti-MTBP (4H9) and anti-cyclin C antibodies. The indicated amino acid positions of MTBP were exchanged against the amino acid, given in one-letter format. Overexp. indicates long exposures in which the stronger signals were saturated. (D) Sequence alignment and conservation of the Cdk8/19-cyclin C binding region of the MTBP middle domain. T-coffee alignment of the amino acid 595–704 fragment of hMTBP that is sufficient to bind Cdk8/19-cyclin C and the corresponding regions from selected vertebrates, branchiostoma and oyster, illustrated using Jalview. Colouring of amino acids indicates similarity according to BLOSUM62 score. BLOSUM62, blocks substitution matrix 62; Cdk8/19, cyclin dependent kinase 8/19; GFP, green fluorescent protein; hMTBP, human MTBP; IP, immunoprecipitation; MTBP, Mdm2 binding protein; Overexp., long exposure in which the stronger signals were saturated; WT, wild-type.
(TIF)

S13 Fig Cyclin C knock-down and Cdk8 binding-deficient MTBP, but not Med12 RNAi, compromise replication in cultured human cells. (A) Immunoblots of whole cell lysates of HeLa-Kyoto cells treated with siCtr or two independent siRNAs against cyclin C (siCycC no. 1 and no. 2) showing that both cyclin C siRNAs specifically knock down cyclin C. All lanes were detected and image processed together from the same immunoblot membrane. (B) HeLa cells show reduced replication speed specifically upon treatment with siCycC. siCtr- and siCycC-treated treated HeLa cells were analysed by BrdU incorporation and flow cytometry to measure BrdU incorporation rates. Replication-specific BrdU signals of S phase cells were normalised to replication signals in siCtr-treated cells. (C) Cyclin C knock-down suppresses replication in U2OS cells. U2OS cells were treated and analysed as described in (B). (D) MTBP knock-down (siMTBP) increases the frequency of fragile chromosomes in HeLa cells. Hela Flp-In T-Rex cells were treated with siCtr or siMTBP. Metaphase spreads were stained with Giemsa. The bar diagram shows an increase in fragile chromosomes in MTBP-depleted cells. This experiment likely underestimates the effect of MTBP depletion on fragile chromosome frequency, because it will be biased towards the subpopulation of cells that are only mildly affected by the MTBP siRNA. This is because the strongly siMTBP-affected subpopulation will not reach mitosis due to their severe replication delay. (E) Expression of Cdk8/19-cyclin C binding-deficient MTBP-ΔP595–P704, but not MTBP-WT, increases fragile chromosome frequency to a similar level as that detected in MTBP-Cdk8bm cells (Figs 6C and S13F and S1 Data). Chromosome fragility of cells treated as in Fig 6A was determined using spreading of metaphase chromosomes. The individual HeLa Flp-In T-Rex cell lines used in this experiment showed no difference in fragile chromosomes in siCtr conditions. (F) HeLa Flp-In T-Rex cells expressing MTBP-Cdk8bm treated with siMTBP, but not those treated with siCtr, show an increase of fragile chromosomes compared with MTBP-WT–expressing cells. Fragile chromosome frequencies of the indicated cell lines were determined using spreading of metaphase chromosomes. MTBP-WT suppresses chromosome fragility to levels found in siCtr cells. (G-H) Med12 knock-down does not result in reduced DNA replication. (G) Immunoblot with whole HeLa cell lysates showing specific knock-down of Med12 by two independent siRNAs (siMed12 no. 1 and no. 2). (H) HeLa cells treated with siMed12 show normal cell cycle profiles with no detectable decrease of replication. HeLa cells treated as in (A) were analysed for PI staining and BrdU incorporation by flow cytometry. The top panels show overlays of BrdU signals of S phase subpopulations of siCtr and siMed12 nos. 1/2–treated cells, indicating no differences in replication speeds. The bottom panels show overlays of PI profiles, indicating only minor differences in cell cycle distribution. BrdU, 5-bromodeoxyuridine; Cdk8, cyclin dependent kinase 8; Cdk8bm, Cdk8 binding mutant; long exp., Med12 signal in siCtr sample in saturated range; Med12, mediator of transcription 12; MTBP, Mdm2 binding protein; no., number; PI, propidium iodide; RNAi, RNA interference; siCtr, control siRNA; siCycC, siRNA against cyclin C; siMed12, siRNA against Med12; siMTBP, MTBP-RNAi; siRNA, small interfering RNA; WT, wild-type.
(TIF)

S14 Fig The core origin firing machinery is conserved between yeast and metazoa. Schematic showing all core factors required for initiation as defined by in vitro reconstitution with purified yeast proteins [16].
(TIF)

S1 Data Flow cytometry gating and raw data of figures involving quantifications. The sheet ‘gating strategy’ shows the gating used for flow cytometry analyses. Cell aggregates were gated out on the basis of Fl2-H and FL2-W dot plots (singlets: gate A). Cell singlets were then shown as PI/BrdU density plots or PI histograms. To quantify G1, S, and G2/M phase subpopulations based on PI histograms, marker regions as shown in (D, E, and F) were used (percent gated events). For analysing DNA replication in detail, PI/BrdU density plots were used to gate the BrdU-positive and BrdU-negative populations, gates N(4) and M(4), respectively. The geometric mean of the Fl1-H channel represented BrdU signal intensity and was used to quantify replication activity and bar graph representations as detailed in the other sheets of S1 Data. Alternatively, the BrdU-positive populations (N(4)) were represented as histogram overlays of the Fl1-H BrdU channel of MTBP (si2/4)- and control (siGL2)-treated cells. Sheets 3C to S13F show the raw data for the figures involving quantifications, namely Figs 3C, 3F, 3G, 4B, 6B–6G and S6B, S7B, S8D, S8F, S9B and S13B–S13F, as indicated in the sheet names. For easy cross-comparison, the graphs shown in the figures are shown for all data sets. BrdU, 5-bromodeoxyuridine; CFS, common fragile site; Exp, experiment; Fl2-H, fluorescence channel 2-hight; FL2-W, fluorescence channel 2-width; FS, fragile site; MTBP, Mdm2 binding protein; PI, propidium iodide; SD, standard deviation; SEM, standard error of the mean; siGL2, control siRNA against firefly luciferase.
(XLSX)

## Abbreviations

ATR/M: ataxia teleangiectasia mutated related/mutated
BLOSUM62: blocks substitution matrix 62
BRCT: breast cancer type 1 susceptibility protein C terminal repeat
BrdU: 5-bromodeoxyuridine
Cdk8bm: Cdk8 binding mutant
Cdc: cell division cycle
Cdk8/19-cyclin C: cyclin-dependent kinase 8/19–cyclinC
Cdt1: Cdc10-dependent transcript
CMG: Cdc45-Mcm2-7-GINS
Dbf4: dumbbell former 4
DDK: Dbf4-dependent kinase
Dpb11: DNA polymerase binding 11
dsDNA: double stranded DNA
FS: fragile metaphase chromosome site
GFP: green fluorescent protein
GINS: go-ichi-ni-san
GST: glutathione S transferase
HA: hemagglutinin
hMTBP: human MTBP
i.p.: intraperitoneally
IP: immunoprecipitation
Mcm: minichromosome maintenance
Med12/13: mediator of transcription subunits 12/13
mSld7: metazoan Sld7
MTBP: Mdm2 binding protein
MTBP-5m: non-Treslin/TICRR binding quintuple point mutant in the S7M-N domain
NLS: nuclear localisation sequence
ORC: origin recognition complex
PCNA: proliferating cell nuclear antigen
PDB: protein data bank
phyre2: protein homology/analogy recognition engine V 2.0
PI: propidium iodide
PPase: phosphatase
pre-LC: preloading complex
pre-RC: prereplicative complex
RecQ4: recombination helicase Q like 4
RNAi: RNA interference
s.c.: subcutaneously
S-CDK: S phase cyclin-dependent kinase
siCtr: control RNAi
siMTBP: MTBP-RNAi
siRNA: small interfering RNA
SIRT1: silent information regulator
Sld3: synthetic lethal with Dpb11 3
STD: Sld3/Treslin domain
S7M-C: Sld7-MTBP C-terminal domain
S7M-N: Sld7-MTBP N-terminal domain
SV40: simian virus 40
TopBP1: Topoisomerase II binding protein 1
TresBD: Treslin/TICRR binding domain
Treslin/TICRR: TopBP1-interacting replication stimulating protein/TopBP1-interacting checkpoint and replication regulator
WT: wild-type

## References

[1] Bell SP, Dutta A. DNA replication in eukaryotic cells. Annu Rev Biochem. 2002;71:333–74. 10.1146/annurev.biochem.71.110601.135425 . 12045100

[2] Diffley JFX. Regulation of early events in chromosome replication. Curr Biol. 2004;14(18):R778–86. 10.1016/j.cub.2004.09.019 . 15380092

[3] Moyer SE, Lewis PW, Botchan MR. Isolation of the Cdc45/Mcm2-7/GINS (CMG) complex, a candidate for the eukaryotic DNA replication fork helicase. Proc Natl Acad Sci U S A. 2006;103(27):10236–41. 10.1073/pnas.0602400103 . 16798881 PMC1482467

[4] Francis LI, Randell JC, Takara TJ, Uchima L, Bell SP. Incorporation into the prereplicative complex activates the Mcm2-7 helicase for Cdc7-Dbf4 phosphorylation. Genes Dev. 2009;23(5):643–54. 10.1101/gad.1759609 . 19270162 PMC2658526

[5] Heller RC, Kang S, Lam WM, Chen S, Chan CS, Bell SP. Eukaryotic Origin-Dependent DNA Replication In Vitro Reveals Sequential Action of DDK and S-CDK Kinases. Cell. 2011;146(1):80–91. Epub 2011/07/07. doi: S0092-8674(11)00654-4 [pii] 10.1016/j.cell.2011.06.012 . 21729781 PMC3204357

[6] Deegan TD, Yeeles JT, Diffley JF. Phosphopeptide binding by Sld3 links Dbf4-dependent kinase to MCM replicative helicase activation. EMBO J. 2016;35(9):961–73. 10.15252/embj.201593552 26912723 PMC4864760

[7] Masumoto H, Muramatsu S, Kamimura Y, Araki H. S-Cdk-dependent phosphorylation of Sld2 essential for chromosomal DNA replication in budding yeast. Nature. 2002;415(6872):651–5. 10.1038/nature713 11807498

[8] Zegerman P, Diffley JFX. Phosphorylation of Sld2 and Sld3 by cyclin-dependent kinases promotes DNA replication in budding yeast. Nature. 2007;445(7125):281–5. Epub 2006/12/15. doi: nature05432 [pii] 10.1038/nature05432 . 17167417

[9] Tanaka S, Umemori T, Hirai K, Muramatsu S, Kamimura Y, Araki H. CDK-dependent phosphorylation of Sld2 and Sld3 initiates DNA replication in budding yeast. Nature. 2007;445(7125):328–32. Epub 2006/12/15. doi: nature05465 [pii] 10.1038/nature05465 . 17167415

[10] Muramatsu S, Hirai K, Tak YS, Kamimura Y, Araki H. CDK-dependent complex formation between replication proteins Dpb11, Sld2, Pol (epsilon}, and GINS in budding yeast. Genes Dev. 2010;24(6):602–12. Epub 2010/03/17. doi: 24/6/602 [pii] 10.1101/gad.1883410 20231317 PMC2841337

[11] Tak YS, Tanaka Y, Endo S, Kamimura Y, Araki H. A CDK-catalysed regulatory phosphorylation for formation of the DNA replication complex Sld2-Dpb11. Embo J. 2006;25(9):1987–96. 10.1038/sj.emboj.7601075 . 16619031 PMC1456926

[12] Merchant AM, Kawasaki Y, Chen Y, Lei M, Tye BK. A lesion in the DNA replication initiation factor Mcm10 induces pausing of elongation forks through chromosomal replication origins in Saccharomyces cerevisiae. Mol Cell Biol. 1997;17(6):3261–71. 9154825 10.1128/mcb.17.6.3261 PMC232179

[13] van Deursen F, Sengupta S, De Piccoli G, Sanchez-Diaz A, Labib K. Mcm10 associates with the loaded DNA helicase at replication origins and defines a novel step in its activation. EMBO J. 2012;31(9):2195–206. Epub 2012/03/22. 10.1038/emboj.2012.69 22433841 PMC3343467

[14] Remus D, Beuron F, Tolun G, Griffith JD, Morris EP, Diffley JFX. Concerted loading of Mcm2-7 double hexamers around DNA during DNA replication origin licensing. Cell. 2009;139(4):719–30. Epub 2009/11/10. doi: S0092-8674(09)01303-8 [pii] 10.1016/j.cell.2009.10.015 . 19896182 PMC2804858

[15] Evrin C, Clarke P, Zech J, Lurz R, Sun J, Uhle S, et al A double-hexameric MCM2-7 complex is loaded onto origin DNA during licensing of eukaryotic DNA replication. Proc Natl Acad Sci U S A. 2009;106(48):20240–5. Epub 2009/11/17. doi: 0911500106 [pii] 10.1073/pnas.0911500106 19910535 PMC2787165

[16] Yeeles JT, Deegan TD, Janska A, Early A, Diffley JF. Regulated eukaryotic DNA replication origin firing with purified proteins. Nature. 2015;519(7544):431–5. 10.1038/nature14285 25739503 PMC4874468

[17] Tanaka T, Umemori T, Endo S, Muramatsu S, Kanemaki M, Kamimura Y, et al Sld7, an Sld3-associated protein required for efficient chromosomal DNA replication in budding yeast. EMBO J. 2011. Epub 2011/04/14. doi: emboj2011115 [pii] 10.1038/emboj.2011.115 . 21487389 PMC3098486

[18] Itou H, Shirakihara Y, Araki H. The quaternary structure of the eukaryotic DNA replication proteins Sld7 and Sld3. Acta Crystallogr D Biol Crystallogr. 2015;71(Pt 8):1649–56. 10.1107/S1399004715010457 . 26249346

[19] Mimura S, Masuda T, Matsui T, Takisawa H. Central role for cdc45 in establishing an initiation complex of DNA replication in Xenopus egg extracts. Genes Cells. 2000;5(6):439–52. . 10886370 10.1046/j.1365-2443.2000.00340.x

[20] Wohlschlegel JA, Dhar SK, Prokhorova TA, Dutta A, Walter JC. Xenopus Mcm10 binds to origins of DNA replication after Mcm2-7 and stimulates origin binding of Cdc45. Mol Cell. 2002;9(2):233–40. . 11864598 10.1016/s1097-2765(02)00456-2

[21] Hashimoto Y, Takisawa H. Xenopus Cut5 is essential for a CDK-dependent process in the initiation of DNA replication. Embo J. 2003;22(10):2526–35. 10.1093/emboj/cdg238 . 12743046 PMC155996

[22] Sangrithi MN, Bernal JA, Madine M, Philpott A, Lee J, Dunphy WG, et al Initiation of DNA replication requires the RECQL4 protein mutated in Rothmund-Thomson syndrome. Cell. 2005;121(6):887–98. 10.1016/j.cell.2005.05.015 . 15960976

[23] Sansam CL, Cruz NM, Danielian PS, Amsterdam A, Lau ML, Hopkins N, et al A vertebrate gene, ticrr, is an essential checkpoint and replication regulator. Genes Dev. 2010;24(2):183–94. Epub 2010/01/19. doi: 24/2/183 [pii] 10.1101/gad.1860310 20080954 PMC2807353

[24] Kumagai A, Shevchenko A, Dunphy WG. Treslin collaborates with TopBP1 in triggering the initiation of DNA replication. Cell. 2010;140(3):349–59. Epub 2010/02/02. doi: S0092-8674(09)01635-3 [pii] 10.1016/j.cell.2009.12.049 20116089 PMC2857569

[25] Sanchez-Pulido L, Diffley JFX, Ponting CP. Homology explains the functional similarities of Treslin/Ticrr and Sld3. Curr Biol. 2010;20(12):R509–10. Epub 2010/07/14. doi: S0960-9822(10)00585-3 [pii] 10.1016/j.cub.2010.05.021 . 20620901

[26] Boos D, Sanchez-Pulido L, Rappas M, Pearl LH, Oliver AW, Ponting CP, et al Regulation of DNA Replication through Sld3-Dpb11 Interaction Is Conserved from Yeast to Humans. Curr Biol. 2011;21(13):1152–7. Epub 2011/06/28. doi: S0960-9822(11)00644-0 [pii] 10.1016/j.cub.2011.05.057 . 21700459

[27] Kumagai A, Shevchenko A, Dunphy WG. Direct regulation of Treslin by cyclin-dependent kinase is essential for the onset of DNA replication. J Cell Biol. 2011;193(6):995–1007. Epub 2011/06/08. doi: jcb.201102003 [pii] 10.1083/jcb.201102003 21646402 PMC3115804

[28] Boos D, Yekezare M, Diffley JF. Identification of a heteromeric complex that promotes DNA replication origin firing in human cells. Science. 2013;340(6135):981–4. Epub 2013/05/25. 10.1126/science.1237448 . 23704573

[29] Kumagai A, Dunphy WG. MTBP, the Partner of Treslin, Contains a Novel DNA-Binding Domain That Is Essential for Proper Initiation of DNA Replication. Mol Biol Cell. 2017 10.1091/mbc.E17-07-0448 . 28877985 PMC5662258

[30] Galbraith MD, Donner AJ, Espinosa JM. CDK8: a positive regulator of transcription. Transcription. 2010;1(1):4–12. 10.4161/trns.1.1.12373 21327159 PMC3035184

[31] Finn RD, Clements J, Arndt W, Miller BL, Wheeler TJ, Schreiber F, et al HMMER web server: 2015 update. Nucleic Acids Res. 2015;43(W1):W30–8. 10.1093/nar/gkv397 25943547 PMC4489315

[32] Wu CH, Apweiler R, Bairoch A, Natale DA, Barker WC, Boeckmann B, et al The Universal Protein Resource (UniProt): an expanding universe of protein information. Nucleic Acids Res. 2006;34(Database issue):D187–91. 10.1093/nar/gkj161 16381842 PMC1347523

[33] Soding J, Biegert A, Lupas AN. The HHpred interactive server for protein homology detection and structure prediction. Nucleic Acids Res. 2005;33(Web Server issue):W244-8. Epub 2005/06/28. doi: 33/suppl_2/W244 [pii] 10.1093/nar/gki408 15980461 PMC1160169

[34] Jones DT. Protein secondary structure prediction based on position-specific scoring matrices. J Mol Biol. 1999;292(2):195–202. 10.1006/jmbi.1999.3091 . 10493868

[35] Gough J. Convergent evolution of domain architectures (is rare). Bioinformatics. 2005;21(8):1464–71. 10.1093/bioinformatics/bti204 . 15585523

[36] Mackay DR, Ullman KS. ATR and a Chk1-Aurora B pathway coordinate postmitotic genome surveillance with cytokinetic abscission. Mol Biol Cell. 2015;26(12):2217–26. 10.1091/mbc.E14-11-1563 25904336 PMC4462940

[37] Agarwal N, Tochigi Y, Adhikari AS, Cui S, Cui Y, Iwakuma T. MTBP plays a crucial role in mitotic progression and chromosome segregation. Cell Death Differ. 2011;18(7):1208–19. Epub 2011/01/29. 10.1038/cdd.2010.189 21274008 PMC3131950

[38] Yeeles JT, Janska A, Early A, Diffley JF. How the Eukaryotic Replisome Achieves Rapid and Efficient DNA Replication. Mol Cell. 2017;65(1):105–16. 10.1016/j.molcel.2016.11.017 27989442 PMC5222725

[39] Devbhandari S, Jiang J, Kumar C, Whitehouse I, Remus D. Chromatin Constrains the Initiation and Elongation of DNA Replication. Mol Cell. 2017;65(1):131–41. 10.1016/j.molcel.2016.10.035 27989437 PMC5256687

[40] Rappas M, Oliver AW, Pearl LH. Structure and function of the Rad9-binding region of the DNA-damage checkpoint adaptor TopBP1. Nucleic Acids Res. 2010. Epub 2010/08/21. doi: gkq743 [pii] 10.1093/nar/gkq743 . 20724438 PMC3017600

[41] Dimitrova DS, Gilbert DM. The spatial position and replication timing of chromosomal domains are both established in early G1 phase. Mol Cell. 1999;4(6):983–93. . 10635323 10.1016/s1097-2765(00)80227-0

[42] Ge XQ, Jackson DA, Blow JJ. Dormant origins licensed by excess Mcm2-7 are required for human cells to survive replicative stress. Genes Dev. 2007;21(24):3331–41. 10.1101/gad.457807 . 18079179 PMC2113033

[43] Natsume T, Muller CA, Katou Y, Retkute R, Gierlinski M, Araki H, et al Kinetochores coordinate pericentromeric cohesion and early DNA replication by Cdc7-Dbf4 kinase recruitment. Mol Cell. 2013;50(5):661–74. 10.1016/j.molcel.2013.05.011 23746350 PMC3679449

[44] Fang D, Lengronne A, Shi D, Forey R, Skrzypczak M, Ginalski K, et al Dbf4 recruitment by forkhead transcription factors defines an upstream rate-limiting step in determining origin firing timing. Genes Dev. 2017;31(23–24):2405–15. 10.1101/gad.306571.117 29330352 PMC5795786

[45] Sansam CG, Pietrzak K, Majchrzycka B, Kerlin MA, Chen J, Rankin S, et al A mechanism for epigenetic control of DNA replication. Genes Dev. 2018;32(3–4):224–9. 10.1101/gad.306464.117 29483155 PMC5859964

[46] Zegerman P, Diffley JFX. Checkpoint-dependent inhibition of DNA replication initiation by Sld3 and Dbf4 phosphorylation. Nature. 2010;467(7314):474–8. Epub 2010/09/14. doi: nature09373 [pii] 10.1038/nature09373 . 20835227 PMC2948544

[47] Lopez-Mosqueda J, Maas NL, Jonsson ZO, Defazio-Eli LG, Wohlschlegel J, Toczyski DP. Damage-induced phosphorylation of Sld3 is important to block late origin firing. Nature. 2010;467(7314):479–83. Epub 2010/09/25. doi: nature09377 [pii] 10.1038/nature09377 . 20865002 PMC3393088

[48] Duch A, Palou G, Jonsson ZO, Palou R, Calvo E, Wohlschlegel J, et al A Dbf4 mutant contributes to bypassing the Rad53-mediated block of origins of replication in response to genotoxic stress. J Biol Chem. 2011;286(4):2486–91. 10.1074/jbc.M110.190843 21098477 PMC3024743

[49] Liu T, Lin YH, Leng W, Jung SY, Zhang H, Deng M, et al A divergent role of the SIRT1-TopBP1 axis in regulating metabolic checkpoint and DNA damage checkpoint. Mol Cell. 2014;56(5):681–95. 10.1016/j.molcel.2014.10.007 25454945 PMC4386886

[50] Wang RH, Lahusen TJ, Chen Q, Xu X, Jenkins LM, Leo E, et al SIRT1 deacetylates TopBP1 and modulates intra-S-phase checkpoint and DNA replication origin firing. Int J Biol Sci. 2014;10(10):1193–202. 10.7150/ijbs.11066 25516717 PMC4261203

[51] Guo C, Kumagai A, Schlacher K, Shevchenko A, Shevchenko A, Dunphy WG. Interaction of Chk1 with Treslin negatively regulates the initiation of chromosomal DNA replication. Mol Cell. 2015;57(3):492–505. 10.1016/j.molcel.2014.12.003 25557548 PMC4321788

[52] Loncle N, Boube M, Joulia L, Boschiero C, Werner M, Cribbs DL, et al Distinct roles for Mediator Cdk8 module subunits in Drosophila development. EMBO J. 2007;26(4):1045–54. 10.1038/sj.emboj.7601566 17290221 PMC1852830

[53] Carrera I, Janody F, Leeds N, Duveau F, Treisman JE. Pygopus activates Wingless target gene transcription through the mediator complex subunits Med12 and Med13. Proc Natl Acad Sci U S A. 2008;105(18):6644–9. 10.1073/pnas.0709749105 18451032 PMC2373359

[54] Steigemann P, Gerlich DW. An evolutionary conserved checkpoint controls abscission timing. Cell Cycle. 2009;8(12):1814–5. . 19471121

[55] Norden C, Mendoza M, Dobbelaere J, Kotwaliwale CV, Biggins S, Barral Y. The NoCut pathway links completion of cytokinesis to spindle midzone function to prevent chromosome breakage. Cell. 2006;125(1):85–98. 10.1016/j.cell.2006.01.045 . 16615892

[56] Fahrenkamp D, de Leur HS, Kuster A, Chatain N, Muller-Newen G. Src family kinases interfere with dimerization of STAT5A through a phosphotyrosine-SH2 domain interaction. Cell Commun Signal. 2015;13:10 10.1186/s12964-014-0081-7 25885255 PMC4350284

[57] Kohler G, Milstein C. Continuous cultures of fused cells secreting antibody of predefined specificity. Nature. 1975;256(5517):495–7. . 1172191 10.1038/256495a0

