# Supplementary figures and images for: The Cdk8/19-cyclin C transcription regulator functions in genome replication through metazoan Sld7
Source: PLoS Biol. 2019 Jan 29;17(1):e2006767. doi: 10.1371/journal.pbio.2006767 (PMC6377148; doi:10.1371/journal.pbio.2006767)

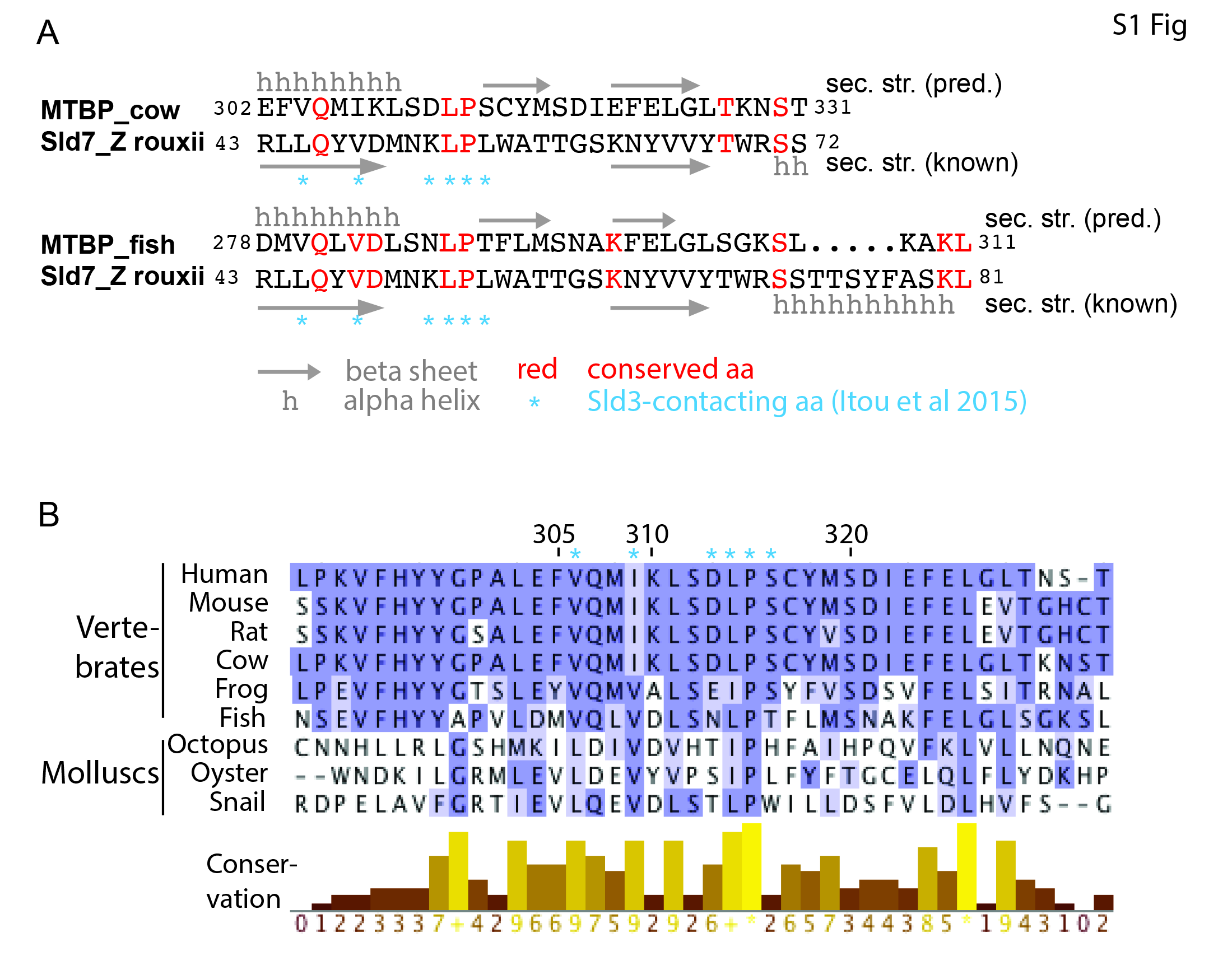

Supplement: S1 Fig — (A) The phyre2 server was queried with MTBP sequences from a selection of metazoan MTBP sequences (human, cow, fish, mouse, frog, octopus). Phyre2 returned alignments between MTBP from cow and fish (Cyprinodon variegatus) with yeast Sld7 with low confidence scores of 11.4% and 13.0%. Numbers are amino acid positions. (B) T-coffee alignment between the indicated MTBPs from selected vertebrates and molluscs, illustrated using Jalview. Colouring of amino acids indicates similarity according to blocks substitution matrix 62 (BLOSUM62) score. Amino acid positions 305, 310, and 320 in hMTBP; blue asterisks, amino acids contacting Sld3 as indicated in (A) [18]; 0–9, +, * indicate relative conservation, with 0 not conserved and * fully conserved. BLOSUM62, blocks substitution matrix 62; hMTBP, human MTBP; MTBP, Mdm2 binding protein; phyre2, protein homology/analogy recognition engine; pred, predicted; Sec. str., secondary structure; Sld7, synthetic lethal with Dpb11 7. (TIF) [file pbio.2006767.s001.tif]

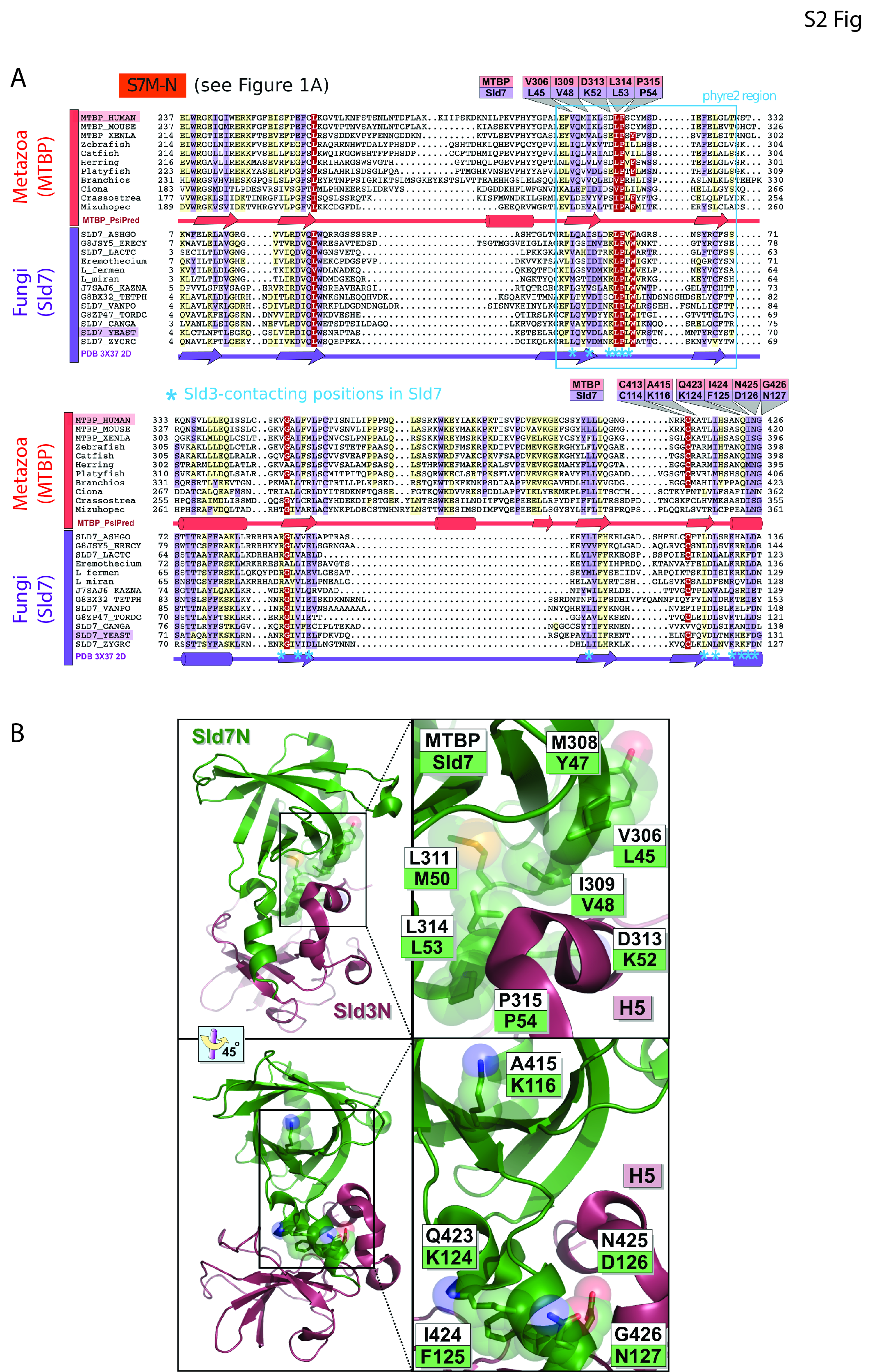

Supplement: S2 Fig — (A) Full sequence alignment, of which parts are shown in Fig 1C, of the S7M-N domains of MTBP and Sld7. In Fig 1C, only the two regions in the S7M of Sld7 that interact with Sld3 are shown. Direct comparison of the N-terminal domain profiles of metazoan MTBP and fungal Sld7 yielded an E-value of 3.0 × 10^−5^ (probability: 78.5%). The phyre2 region and the region containing the second cluster of Sld3-interacting amino acids (C413–G426) are indicated. Asterisks mark the positions of Sld3-interacting amino acids in yeast Sld7 and the corresponding positions in MTBP. (B) Critical Treslin/TICRR-binding amino acids in MTBP map to Sld7 residues that directly contact Sld3 in the crystal structure of the Sld7-Sld3 dimer [18]. MTBP, Mdm2 binding protein; Sld7, synthetic lethal with Dpb11 7; S7M-N, Sld7-MTBP N-terminal domain; TICRR, TopBP1 interacting checkpoint and replication regulator. (TIF) [file pbio.2006767.s002.tif]

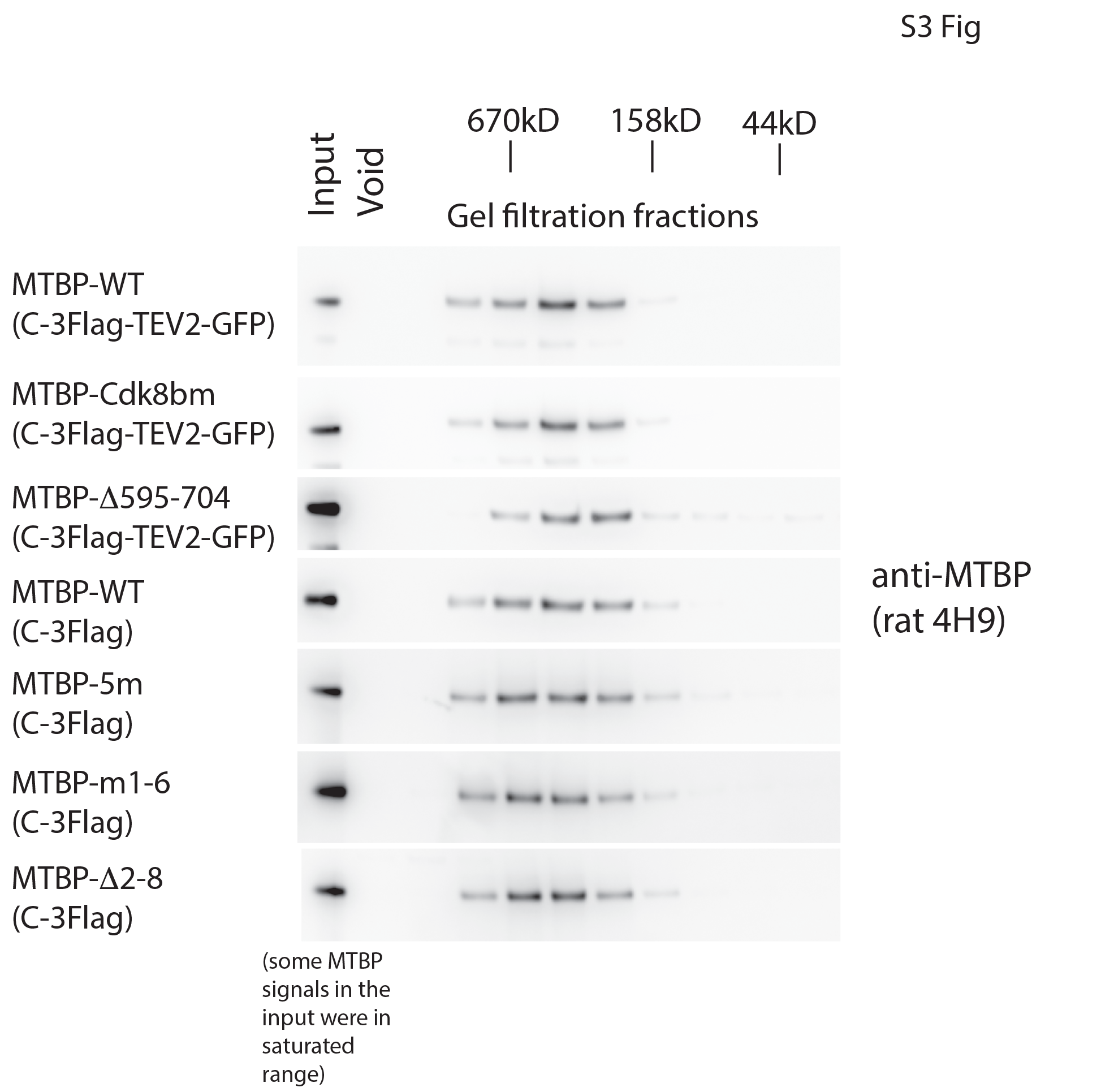

Supplement: S3 Fig — Flag-tagged MTBP-WT and indicated mutant MTBP proteins were isolated by Flag IP and Flag peptide elution from lysates of transiently transfected, and therefore highly overexpressing, 293T cells. Separation by gel filtration using a Superdex 200 column (3.2/300; 2.4 mL) followed. All MTBP versions eluted in distinct peaks, and similar fractions of the input were recovered, indicating stable folding. MTBP-Δ595–704 eluted slightly later from the column due to its smaller size. MTBP, Mdm2 binding protein; WT, wild-type. (TIF) [file pbio.2006767.s003.tif]

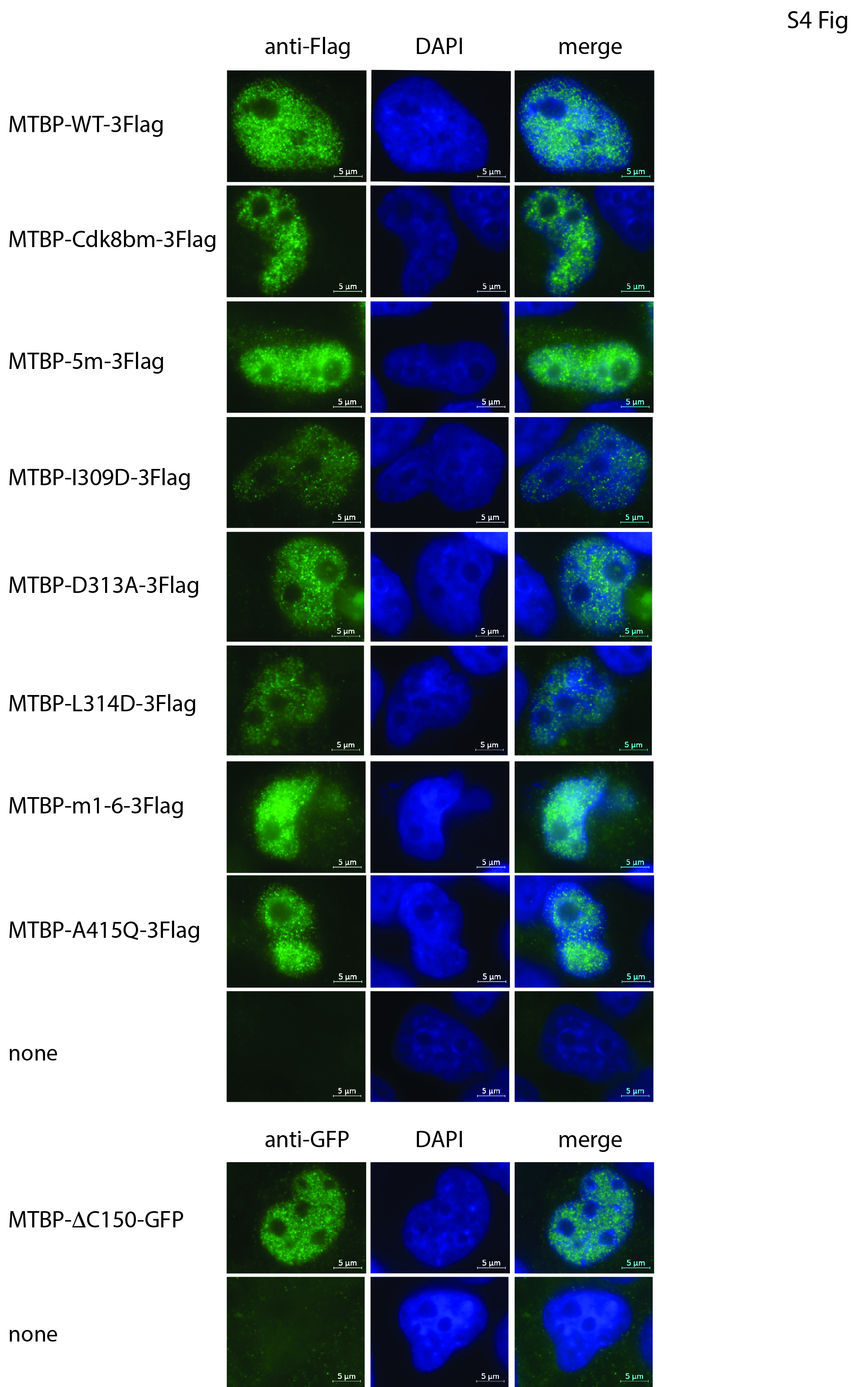

Supplement: S4 Fig — Hela Flp-In cells were transiently transfected with the indicated Flag- or GFP-tagged MTBP-WT and mutants, and then immune-stained with anti-Flag or anti-GFP, as indicated. ‘none’ indicates nontransfected cells. dsDNA staining (Hoechst 33258) served to mark the nuclear DNA. dsDNA, double stranded DNA; GFP, green fluorescent protein; MTBP, Mdm2 binding protein; WT, wild-type. (TIF) [file pbio.2006767.s004.tif]

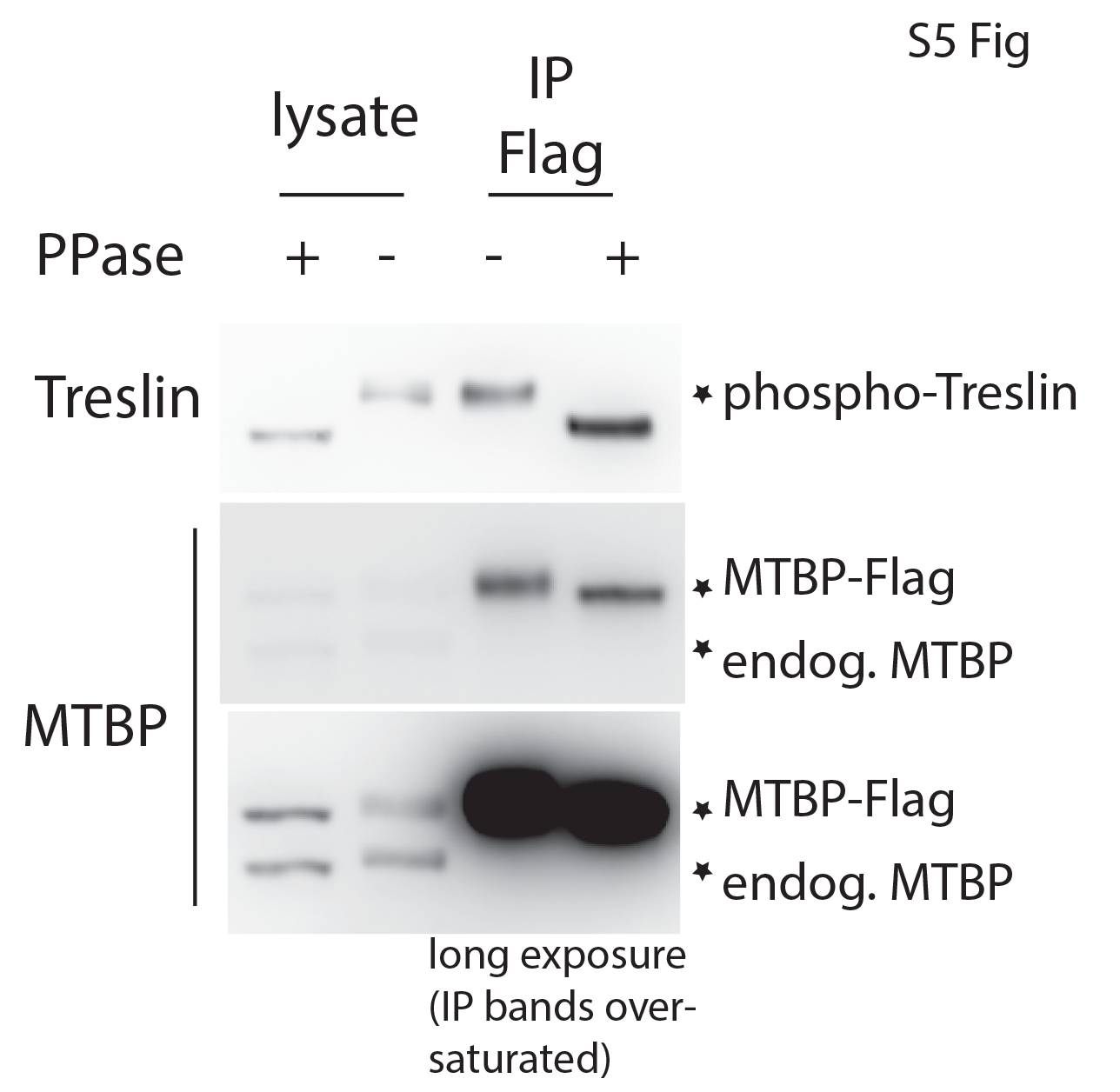

Supplement: S5 Fig — Cell lysates of Hela cells (in 20 mM HEPES, 150 mM NaCl, 10% glycerol, complete EDTA-free protease inhibitor cocktail, 0.1% Triton X-100, 2 mM 2-mercaptoethanol expressing MTBP-WT-3Flag) were treated with lambda PPase (8,000 units in 1mL lysate; NEB P0753) or buffer according to the manufacturer’s instructions. Lysates were then used for Flag IPs with 40 uL slurry of M2 anti-Flag magnetic beads (M8823, Sigma) to isolate MTBP-Flag and associated Treslin/TICRR. Lysates and IPs were separated on a 3%–8% Tris-acetate Criterion gel (BioRad) for optimal resolution. Immunoblotting for Treslin/TICRR and MTBP showed that the gel mobility of Treslin/TICRR shifts in PPase treatment in lysates, indicating phosphorylation. In the absence of PPase, Treslin/TICRR in MTBP-bound Treslin/TICRR showed indistinguishable running behaviour from Treslin/TICRR in lysates, indicating phosphorylation of MTBP-bound Treslin/TICRR. IP, immunoprecipitation; MTBP, Mdm2 binding protein; PPase, phosphatase; TICRR, TopBP1 interacting checkpoint and replication regulator; WT, wild-type. (TIF) [file pbio.2006767.s005.tif]

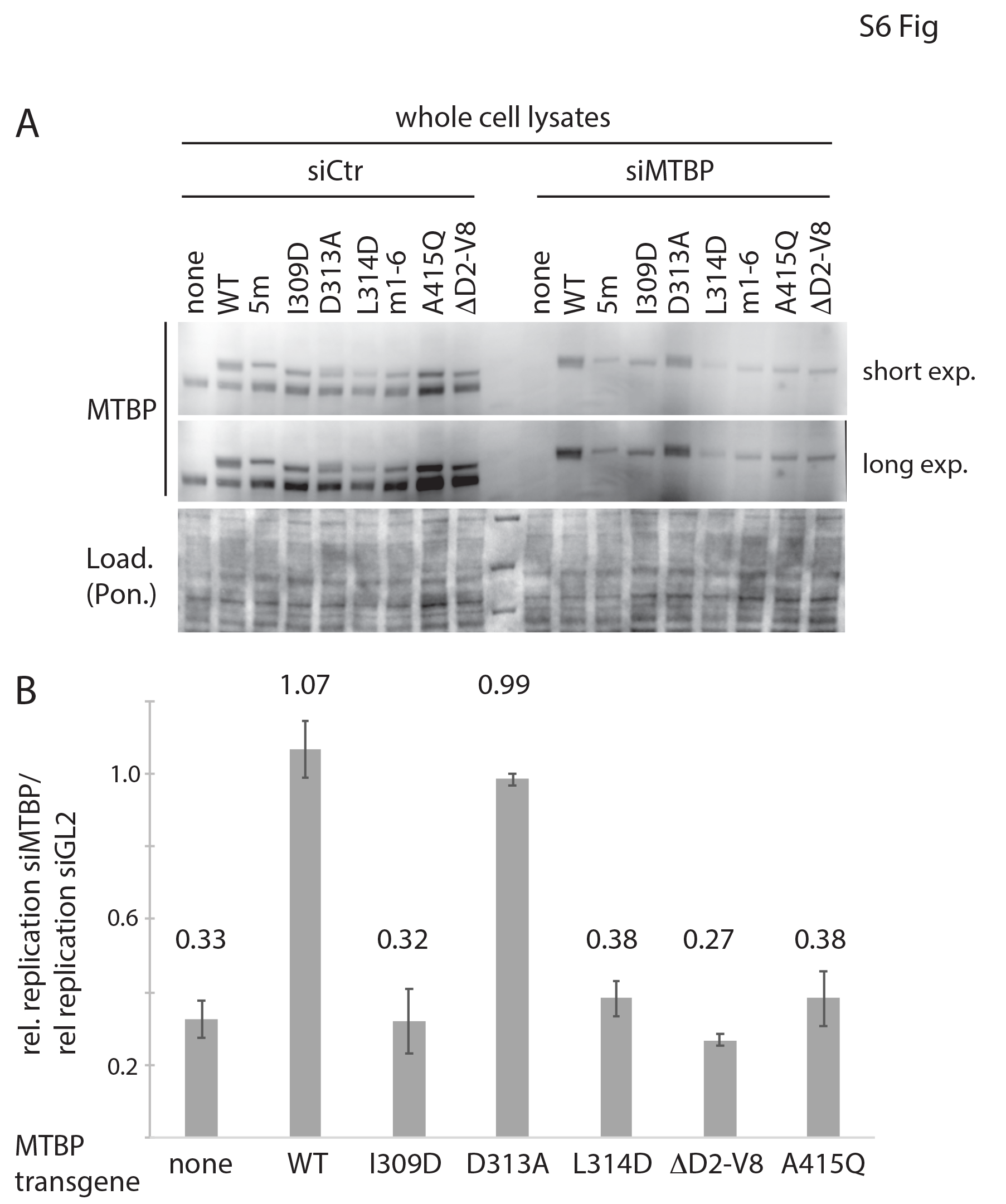

Supplement: S6 Fig — (A) Immunoblot showing MTBP levels in lysates of siCtr of siMTBP-treated cells expressing siRNA-resistant MTBP-WT-3Flag or mutants carrying the indicated single amino acid exchanges. The combination mutants 5m and m1–6, from which the single mutants were derived, are shown for comparison. In the long exposure, the stronger signals in siCtr lysates were in the saturated range. (B) Replication was quantified using BrdU incorporation and flow cytometry after replacing endogenous MTBP with RNAi-resistant MTBP-WT or mutants in stable Hela Flp-In cell lines. I309D, D313A, and L314D are single point mutations that were also mutated in the MTBP-5m combination mutant. I309D and L314D were defective in Treslin/TICRR binding, whereas D313A bound Treslin/TICRR like MTBP-WT (Fig 2A). A415Q, also mutated in the m1–6 combination mutant, was Treslin/TICRR-binding deficient, as was MTBP-ΔD2-V8 (Fig 2B). To show that the cells expressing Treslin/TICRR nonbinding mutants showed similarly low replication activity as siMTBP-treated cells without a transgene, the data were not normalised to no-transgene cells for this graph. Error bars: SEM from three independent experiments. BrdU, 5-bromodeoxyuridine; m, point mutation; MTBP, Mdm2 binding protein; RNAi, RNA interference; siCtr, control RNAi; siMTBP, MTBP-RNAi; siRNA, small interfering RNA; S7M-N, Sld7-MTBP N-terminal domain; TICRR, TopBP1 interacting checkpoint and replication regulator; WT, wild-type. (TIF) [file pbio.2006767.s006.tif]

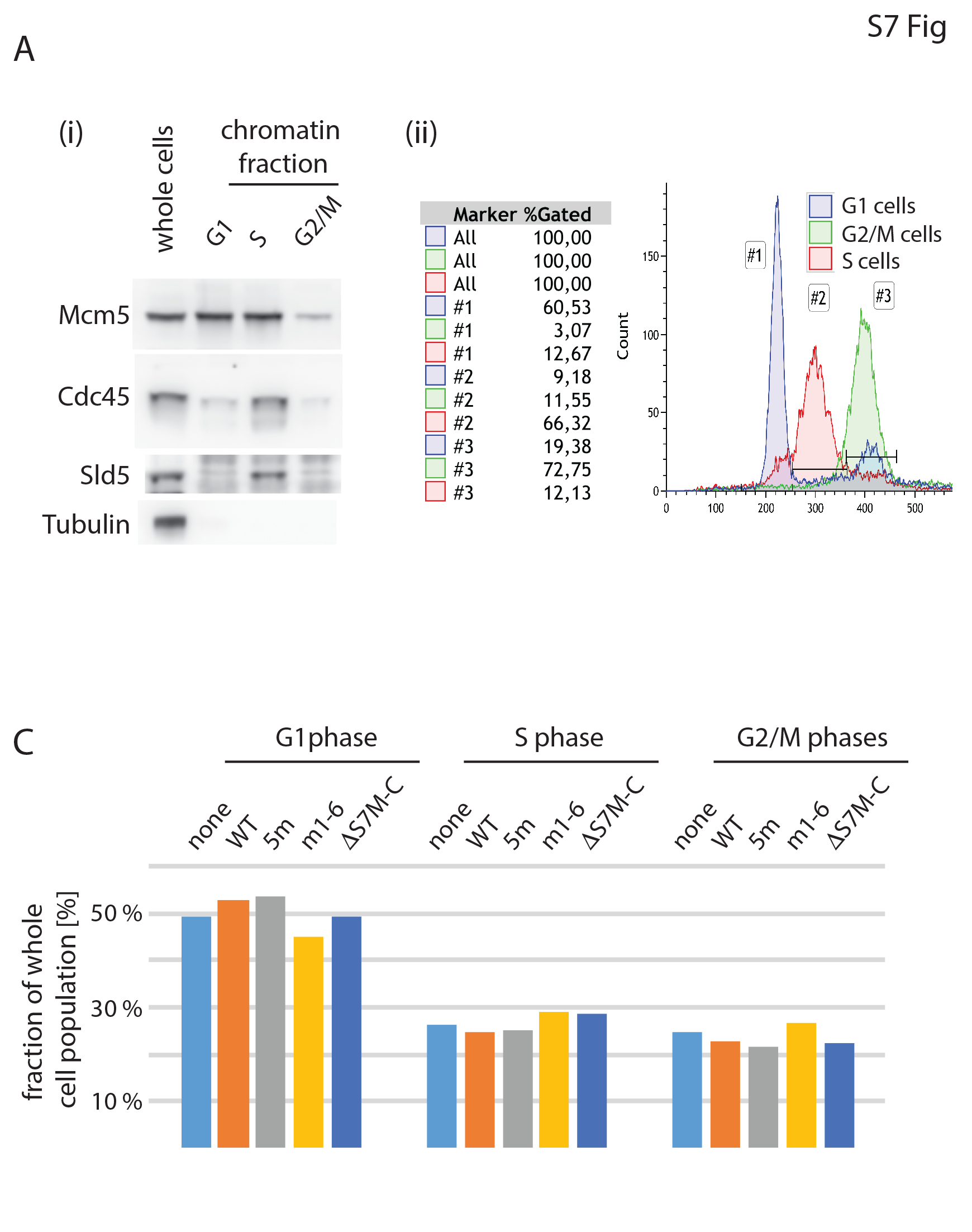

Supplement: S7 Fig — (A) Origin licensing and firing can be assessed by immunoblotting of chromatin isolated from cells. For (i), chromatin was isolated from cells synchronised in mitosis, in G1 or S phase, using thymidine arrest and release for 3 h (S phase cells), 10 h (G2/M), or 14 h (G1). Mcm5 signals present in G1 chromatin, but less in mitosis, showed that licensing can be monitored. Cdc45 and Sld5 (GINS) were detected specifically on S phase chromatin, confirming these are adequate markers for origin firing. (ii) Shows PI-based cell cycle analysis by flow cytometry of samples used in (i). (B) Flow cytometry of the PI-stained cells described in Fig 3D showed that the cell cycle distribution was largely unchanged under the experimental conditions. Cdc45, cell division cycle 45; GINS, go-ichi-ni-san; Mcm5, minichromosome maintenance 5; PI, propidium iodide; Sld5, synthetic lethal with Dpb11 5. (TIF) [file pbio.2006767.s007.tif]

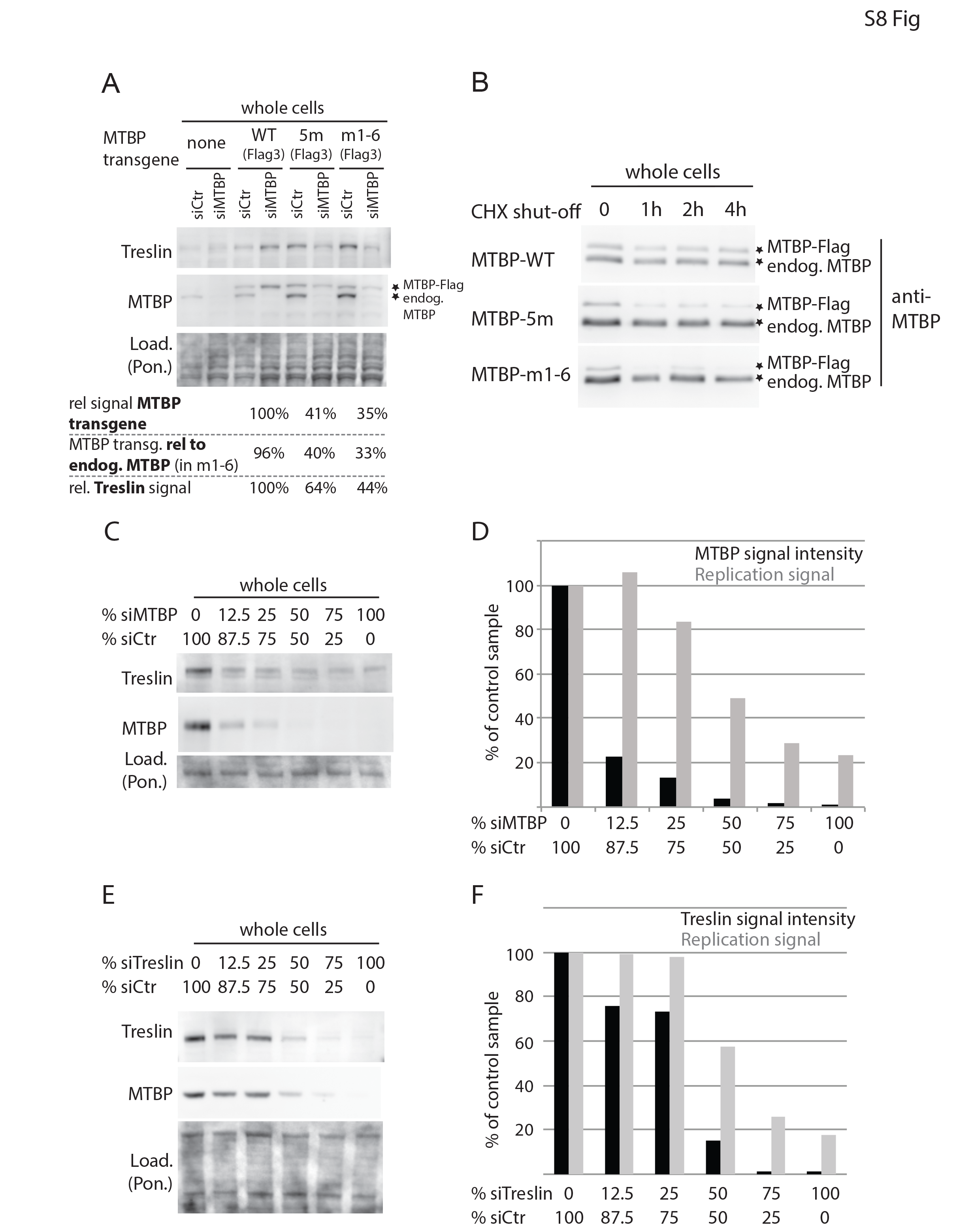

Supplement: S8 Fig — (A) Quantification of immunoblot signals of samples shown in Fig 3A shows that Treslin/TICRR binding deficient MTBP mutants have only moderately decreased levels of MTBP and Treslin/TICRR. (B) Treslin binding-deficient MTBP is less stable than MTBP-WT. HeLa Flp-In T-Rex cells expressing MTBP-3×Flag-WT or the indicated Treslin/TICRR binding-deficient mutants were doxycycline induced overnight before shutoff of gene expression by doxycycline withdrawal and addition of 100 μg/mL cycloheximide for the indicated times. Whole cell lysates were then immunoblotted with anti-MTBP antibodies (4H9). The Treslin/TICRR binding deficient mutants decreased in levels within 1 h. In contrast, endogenous MTBP and MTBP-WT-3×Flag were largely stable for 4 h. (C,D) Strong down-regulation of MTBP is required to suppress replication. HeLa Flp-In T-Rex control cells were treated with increasing concentrations of siMTBP. Treslin/TICRR levels decrease moderately in MTBP-depleted cells. Whole cell lysates were immunoblotted to assess relative levels of MTBP and Treslin/TICRR (C). (D) Shows quantification of the immunoblot signals shown in (C) and of replication levels, as assessed by BrdU incorporation and flow cytometry. (D) Indicates that MTBP signals need to decrease by roughly 90% to achieve significant reduction of replication in these cells. (E,F) Strong down-regulation of Treslin/TICRR is required to suppress replication. MTBP levels strongly decline upon Treslin/TICRR depletion. The experiment described in (C, D) was repeated with siRNA against Treslin/TICRR. BrdU, 5-bromodeoxyuridine; MTBP, Mdm2 binding protein; siMTBP, MTBP-RNAi; siRNA, small interfering RNA; TICRR, TopBP1 interacting checkpoint and replication regulator; WT, wild-type. (TIF) [file pbio.2006767.s008.tif]

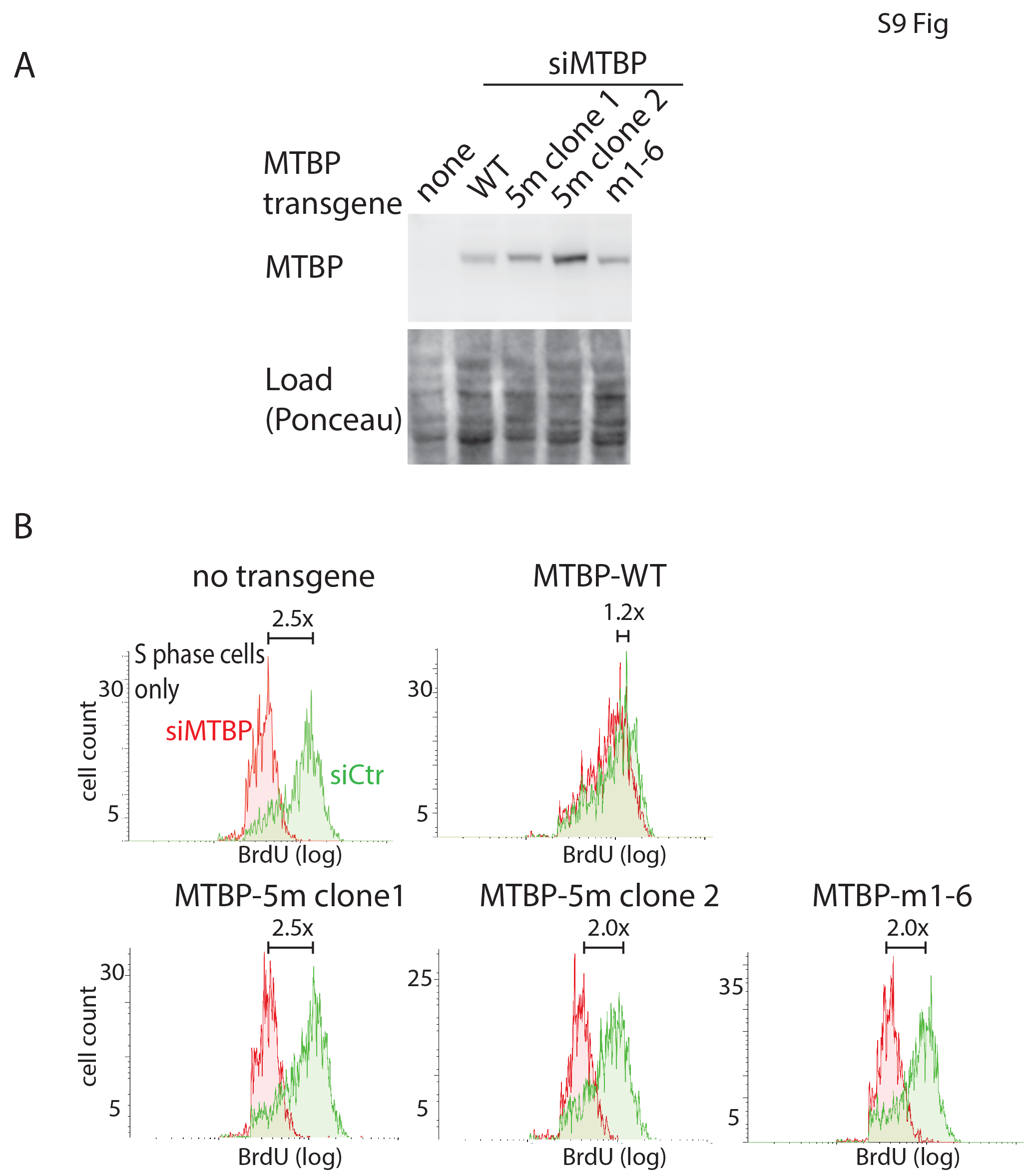

Supplement: S9 Fig — Hela Kyoto cell lines with stably integrated siMTBP-resistant MTBP-WT-Flag-GFP, MTBP-5m-Flag-GFP (clones 1 and 2), or MTBP-m1–6-Flag-GFP in pIRES-puro3 were RNAi treated with siCtr or siMTBP. (A) Immunoblot showing the relative levels of the transgenes. (B) Replication was measured by BrdU incorporation and flow cytometry. S phase subpopulations of siCtr- and siMTBP-treated samples were overlaid and the fold difference of BrdU signal calculated (1.2–2.5×; note that numbers are not directly comparable with Fig 3C, F because of the different Hela cell lines used). In contrast to MTBP-WT, the -5m and -m1–6 mutants support DNA replication poorly despite equal (5m clone 1, m1–6) or higher (5m clone 2) expression levels. BrdU, 5-bromodeoxyuridine; GFP, green fluorescent protein; m, point mutation; MTBP, Mdm2 binding protein; pIRES, plasmid named internal ribosomal entry site; puro3, puromycin 3; RNAi, RNA interference; siCtr, control RNAi; siMTBP, MTBP-RNAi; TICRR, TopBP1 interacting checkpoint and replication regulator; WT, wild-type. (TIF) [file pbio.2006767.s009.tif]

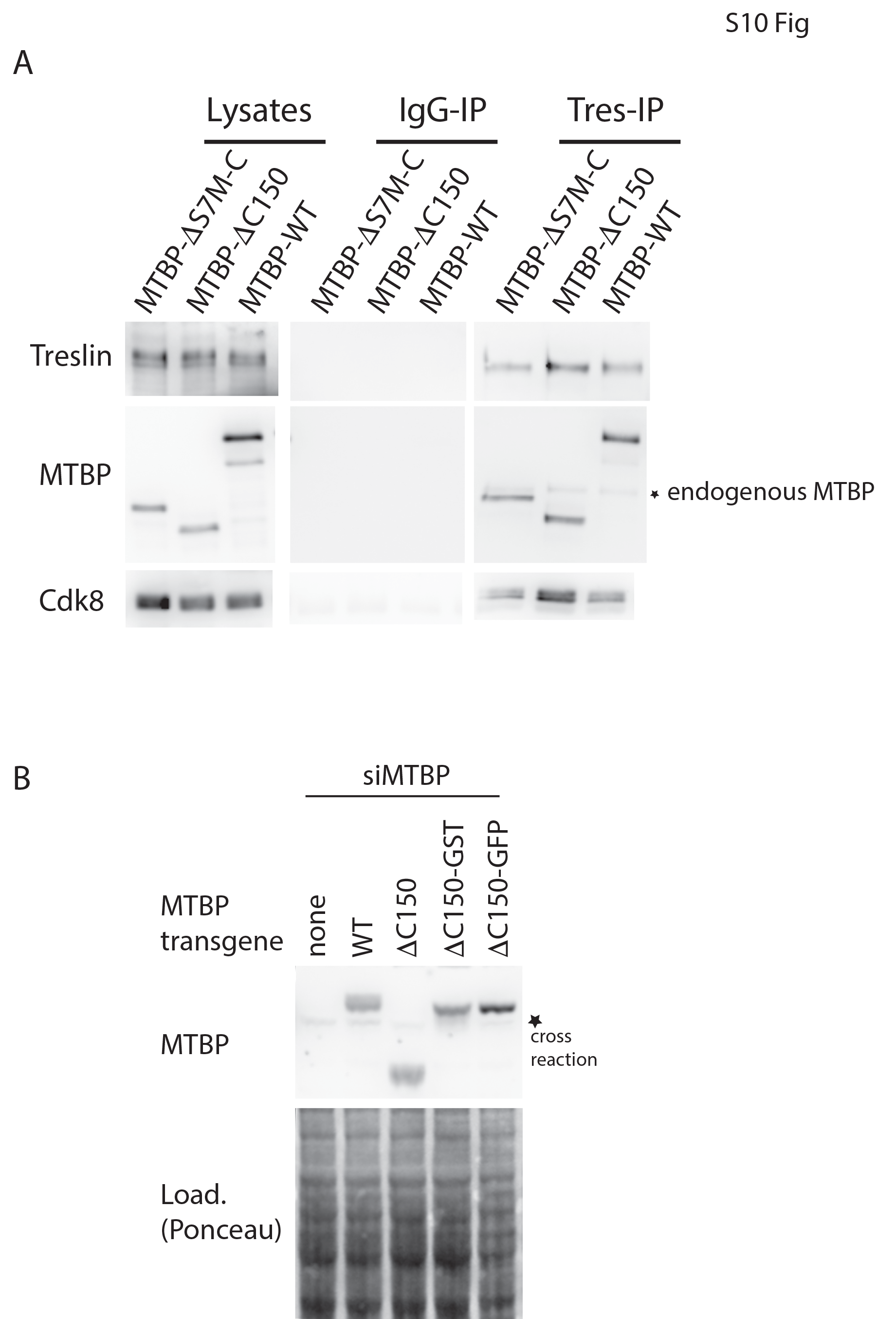

Supplement: S10 Fig — (A) Mutants in the S7M-C domain of MTBP are proficient in Treslin binding. Native lysates of HeLa Flp-In T-Rex cells expressing MTBP-WT or mutants lacking the last 81 (ΔS7M-C) or 150 (ΔC150) amino acids were used for IP of endogenous Treslin using control IgGs (IgG-IP) or rabbit anti-Treslin-970-1400 antibodies (Tres-IP). Lysates and bead-bound material were then analysed in immunoblots using anti-MTBP and anti-Treslin antibodies. B) siMTBP-treated Hela Flp-In cell lines MTBP-WT-3Flag, MTBPΔC150, MTBPΔC150-GST, and MTBPΔC150-GFP were lysed in SDS sample buffer and tested by immunoblotting with anti-hMTBP (83) for expression levels of the transgenes. Cdk8, cyclin dependent kinase 8; GFP, green fluorescent protein; GST, glutathione S transferase; hMTBP, human MTBP; IgG, immunoglobulin; IP, immunoprecipitation; MTBP, Mdm2 binding protein; siMTBP, MTBP-RNAi; S7M-C, Sld7-MTBP C-terminal domain; WT, wild-type. (TIF) [file pbio.2006767.s010.tif]

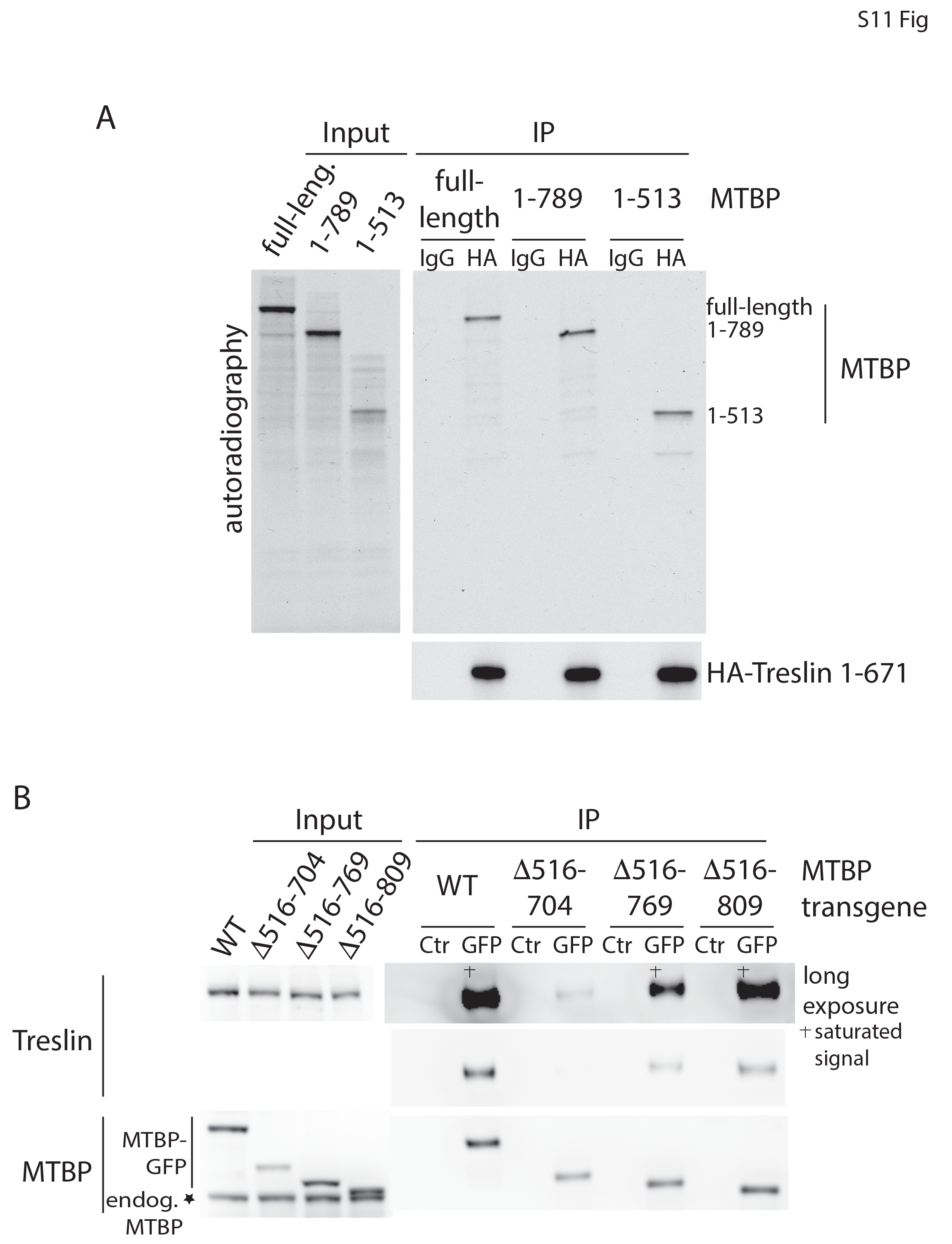

Supplement: S11 Fig — (A) Treslin/TICRR binding activities of N-terminal MTBP fragments. hMTBP, full length, or the indicated fragments were translated in SP6 reticulocyte lysates (Promega L2080) in the presence of 35S-methionine, and HA-Treslin/TICRR-1-671 was translated in lysates without radioactive methionine. MTBP and Treslin/TICRR lysates were mixed, and IPs with unspecific IgG or anti-HA antibodies were made. Lysates (Inputs) and bead-bound material (IP) were analysed by autoradiography and anti-HA immunoblotting. (B) Binding of mSld7s to Treslin/TICRR. Lysates of Fig 4A were used for IPs with GFP nanobodies or mock-coupled control beads, and then analysed for bead-bound MTBP and Treslin/TICRR by immunoblotting with anti-MTBP (4H9) and anti-Treslin (148). GFP, green fluorescent protein; HA, hemagglutinin; hMTBP, human MTBP; IgG, immunoglobulin; IP, immunoprecipitation; mSld7, metazoan Sld7; MTBP, Mdm2 binding protein; SP6, SP6 virus; TICRR, TopBP1 interacting checkpoint and replication regulator. (TIF) [file pbio.2006767.s011.tif]

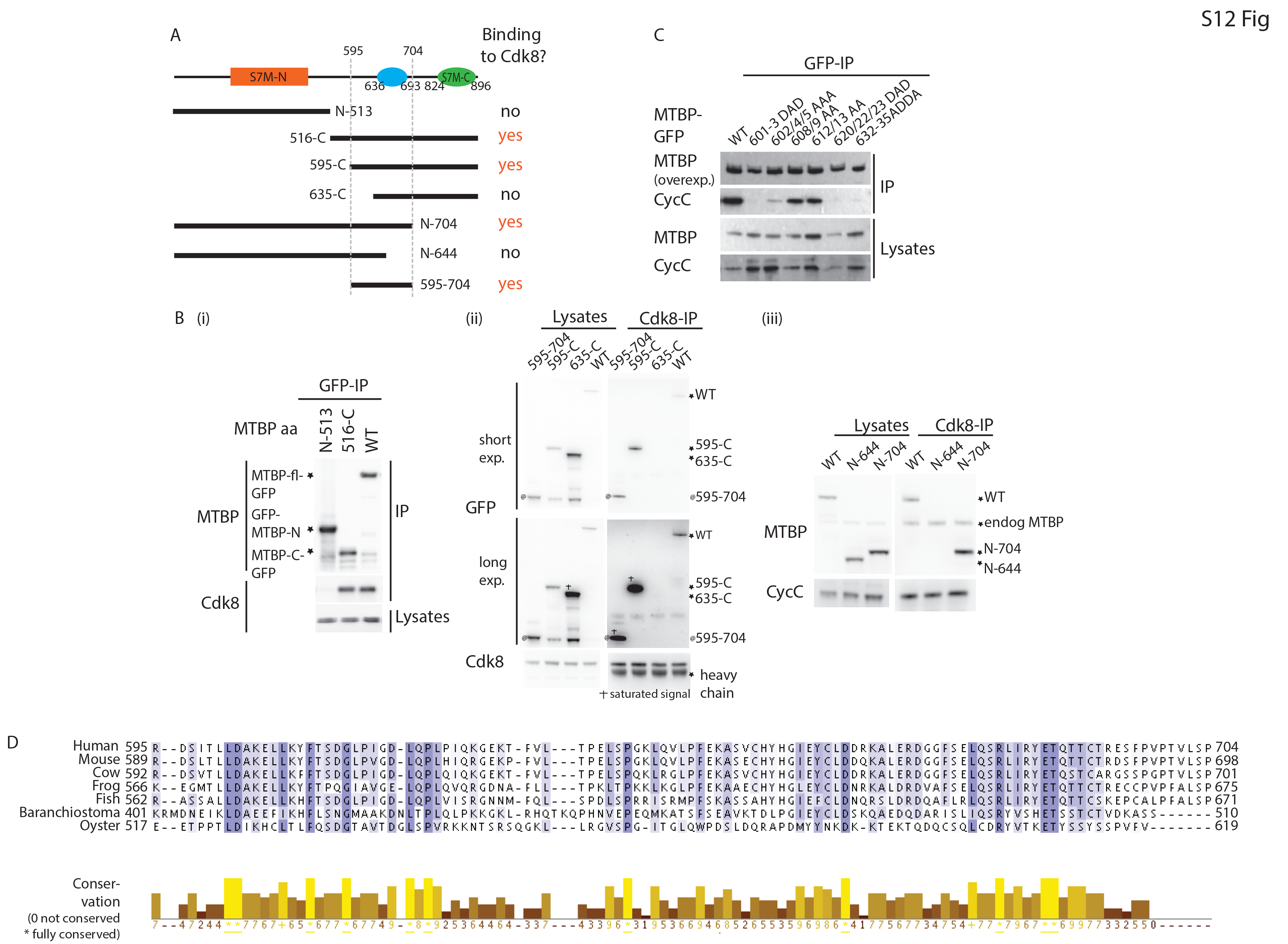

Supplement: S12 Fig — (A) Schematic summarising MTBP fragment-based Cdk8/19-cyclin C binding studies shown in (B). (B) MTBP-WT or N- or C-terminal fragments of MTBP containing the indicated amino acids (aa) were transiently transfected into 293T cells. Subsequent IP from native lysates with the anti-GFP (i) or anti-Cdk8 (ii, iii) antibodies were analysed by immunoblotting using anti-MTBP (4H9), anti-Cdk8, anti-GFP, or anti-cyclin C antibodies. Overexp. indicates long exposures in which the stronger signals were saturated. (C) MTBP-Flag-GFP-WT or the indicated point mutants in the Cdk8/19-cyclin C binding region mapped in (A,B) were tested for interaction with endogenous Cdk8/19-cyclin C using transient transfection of MTBP into 293T cells and anti-GFP IP. Subsequent immunoblots were analysed with anti-MTBP (4H9) and anti-cyclin C antibodies. The indicated amino acid positions of MTBP were exchanged against the amino acid, given in one-letter format. Overexp. indicates long exposures in which the stronger signals were saturated. (D) Sequence alignment and conservation of the Cdk8/19-cyclin C binding region of the MTBP middle domain. T-coffee alignment of the amino acid 595–704 fragment of hMTBP that is sufficient to bind Cdk8/19-cyclin C and the corresponding regions from selected vertebrates, branchiostoma and oyster, illustrated using Jalview. Colouring of amino acids indicates similarity according to BLOSUM62 score. BLOSUM62, blocks substitution matrix 62; Cdk8/19, cyclin dependent kinase 8/19; GFP, green fluorescent protein; hMTBP, human MTBP; IP, immunoprecipitation; MTBP, Mdm2 binding protein; Overexp., long exposure in which the stronger signals were saturated; WT, wild-type. (TIF) [file pbio.2006767.s012.tif]

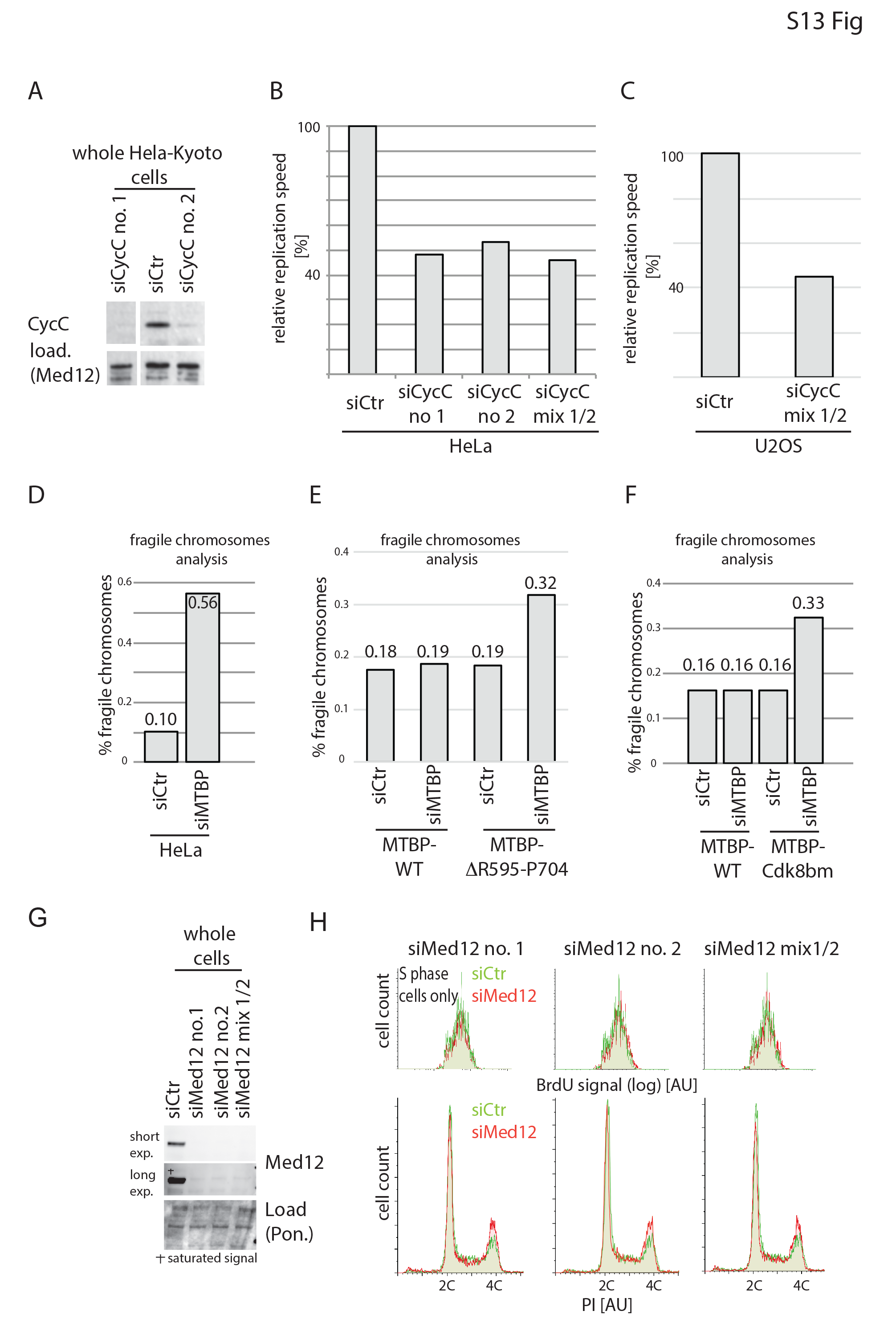

Supplement: S13 Fig — (A) Immunoblots of whole cell lysates of HeLa-Kyoto cells treated with siCtr or two independent siRNAs against cyclin C (siCycC no. 1 and no. 2) showing that both cyclin C siRNAs specifically knock down cyclin C. All lanes were detected and image processed together from the same immunoblot membrane. (B) HeLa cells show reduced replication speed specifically upon treatment with siCycC. siCtr- and siCycC-treated treated HeLa cells were analysed by BrdU incorporation and flow cytometry to measure BrdU incorporation rates. Replication-specific BrdU signals of S phase cells were normalised to replication signals in siCtr-treated cells. (C) Cyclin C knock-down suppresses replication in U2OS cells. U2OS cells were treated and analysed as described in (B). (D) MTBP knock-down (siMTBP) increases the frequency of fragile chromosomes in HeLa cells. Hela Flp-In T-Rex cells were treated with siCtr or siMTBP. Metaphase spreads were stained with Giemsa. The bar diagram shows an increase in fragile chromosomes in MTBP-depleted cells. This experiment likely underestimates the effect of MTBP depletion on fragile chromosome frequency, because it will be biased towards the subpopulation of cells that are only mildly affected by the MTBP siRNA. This is because the strongly siMTBP-affected subpopulation will not reach mitosis due to their severe replication delay. (E) Expression of Cdk8/19-cyclin C binding-deficient MTBP-ΔP595–P704, but not MTBP-WT, increases fragile chromosome frequency to a similar level as that detected in MTBP-Cdk8bm cells (Figs 6C and S13F and S1 Data). Chromosome fragility of cells treated as in Fig 6A was determined using spreading of metaphase chromosomes. The individual HeLa Flp-In T-Rex cell lines used in this experiment showed no difference in fragile chromosomes in siCtr conditions. (F) HeLa Flp-In T-Rex cells expressing MTBP-Cdk8bm treated with siMTBP, but not those treated with siCtr, show an increase of fragile chromosomes compared with MTBP-WT–expressing [file pbio.2006767.s013.tif]

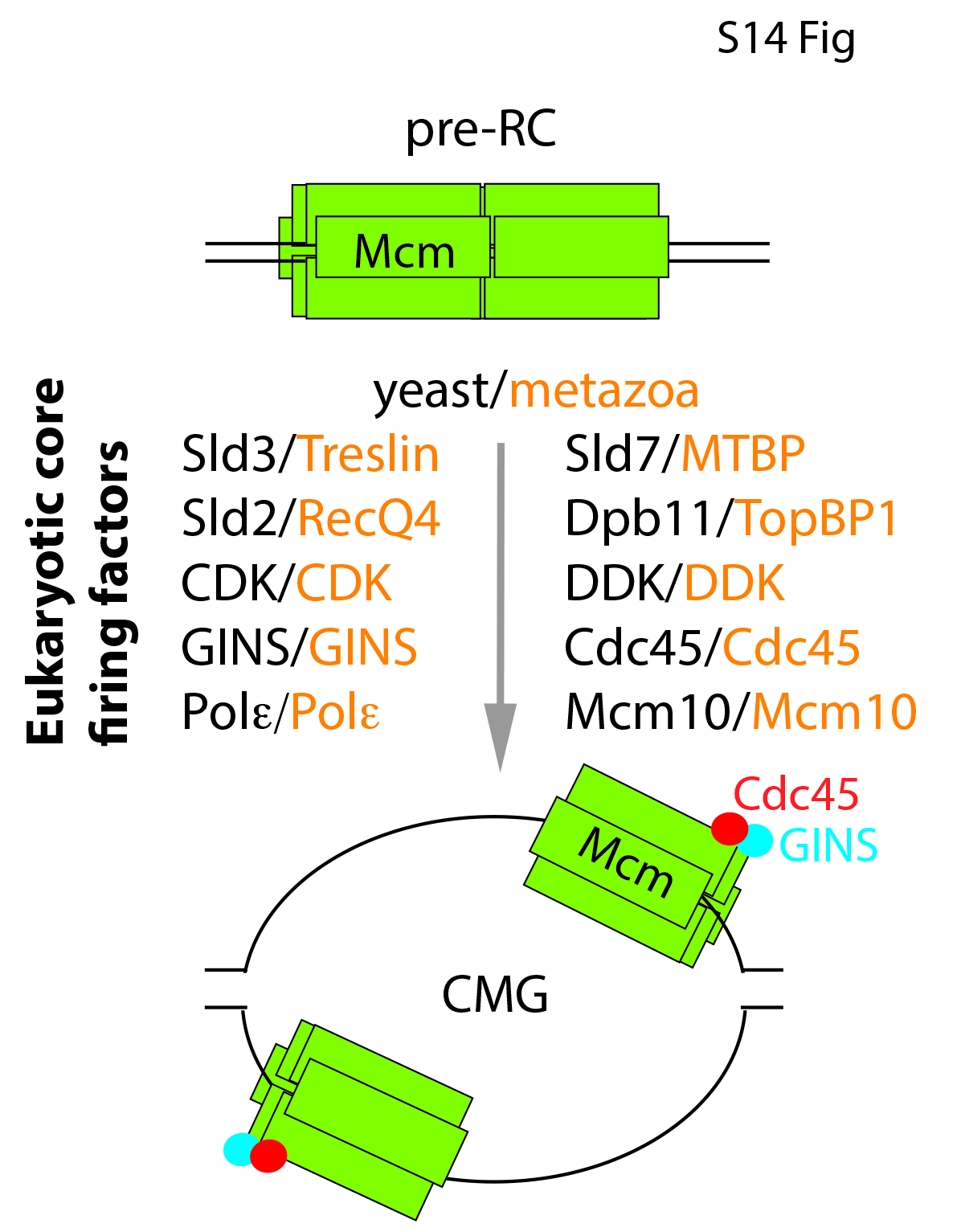

Supplement: S14 Fig — Schematic showing all core factors required for initiation as defined by in vitro reconstitution with purified yeast proteins [16]. (TIF) [file pbio.2006767.s014.tif]
